# Tandem redox mediator/Ni(ii) trihalide complex photocycle for hydrogen evolution from HCl†
†Electronic supplementary information (ESI) available: Experimental procedures and time-dependent photochemical data; transient absorption spectroscopy and electrochemical experimental details. CCDC 992218–992221. For ESI and crystallographic data in CIF or other electronic format see DOI: 10.1039/c4sc02357a



**DOI:** 10.1039/c4sc02357a

**Published:** 2014-10-08

**Authors:** Seung Jun Hwang, David C. Powers, Andrew G. Maher, Daniel G. Nocera

**Affiliations:** a Department of Chemistry and Chemical Biology , 12 Oxford Street , Cambridge , MA 02138-2902 , USA . Email: dnocera@fas.harvard.edu; b Department of Chemistry , Massachusetts Institute of Technology , 77 Massachusetts Avenue , Cambridge , MA 02139-4307 , USA

## Abstract

Photoactivation of M–X bonds is a challenge for photochemical HX splitting, particularly with first-row transition metal complexes because of short intrinsic excited state lifetimes. Herein, we report a tandem H_2_ photocycle based on combination of a non-basic photoredox phosphine mediator and nickel metal catalyst. Synthetic studies and time-resolved photochemical studies have revealed that phosphines serve as photochemical H-atom donors to Ni(ii) trihalide complexes to deliver a Ni(i) centre. The H_2_ evolution catalytic cycle is closed by sequential disproportionation of Ni(i) to afford Ni(0) and Ni(ii) and protolytic H_2_ evolution from the Ni(0) intermediate. The results of these investigations suggest that H_2_ photogeneration proceeds by two sequential catalytic cycles: a photoredox cycle catalyzed by phosphines and an H_2_-evolution cycle catalyzed by Ni complexes to circumvent challenges of photochemistry with first-row transition metal complexes.

## Abstract 2

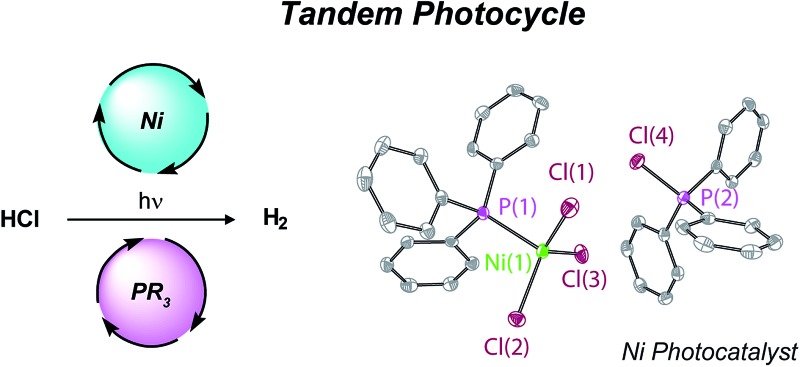
The challenge that short excited state lifetimes of first-row transition metal complexes present to the photoactivation of M–X bonds has been overcome with a phosphine mediator coupled to a nickel metal catalyst.

## Introduction

The photochemical splitting of hydrohalic acids (HX) into H_2_ and X_2_ is an approach to solar fuel synthesis^1^ that stores a comparable amount of energy to water splitting. In addition to the similar energy densities implicit in HX and H_2_O splitting chemistries, HX splitting mandates management of only two electrons and two protons, whereas H_2_O splitting requires management of four protons and four electrons.^2–5^ Photocatalytic HX splitting requires accomplishing multielectron photochemical reactions to activate strong M–X bonds. Typically, photoreduction has been the limiting step in HX splitting photocatalysis and often X_2_ elimination requires the use of chemical traps for evolved halogen equivalents.^6–17^ Attempts at promoting HX splitting with first-row transition metal complexes are attractive given that these metals are typically earth abundant, but have been largely unsuccessful. The challenge of using first-row metal complexes as HX splitting photocatalysts is that photochemical activation of Ni(i) or Ni(ii) halides frequently does not lead to photoreduction reactions,^18,19^ likely due to the short excited state lifetimes of first-row transition metal complexes.^20–22^

To overcome the short excited state lifetimes typical of first-row complexes, we have pursued a photoredox strategy for H_2_ evolution from HCl in which the photochemistry and H_2_ evolution roles are separated between a photoredox mediator and a hydrogen-evolution catalyst, respectively.^23^ We were attracted to this strategy because it does not rely on molecular excited states of first-row metal complexes. In our first foray into photoredox catalysis for H_2_ evolution, we employed bipyridines as photoredox mediators and Ni polypyridyl complexes as H_2_ evolution catalysts. H-atom abstraction (HAA) by the excited state of the bipyridine afforded a pyridinyl radical, which engaged with a Ni(ii) halide complex to generate a Ni(i) intermediate *via* halogen radical abstraction. The resulting Ni(i) complex underwent disproportionation to a Ni(ii) complex and a Ni(0) species, which subsequently engaged in protolytic H_2_ evolution. While these efforts demonstrated a synthetic cycle for H_2_ evolution, H_2_-evolution catalysis was not observed because the basic bipyridyl photoredox mediator was passivated in the presence of HCl.

To address the challenges of photoredox catalysis for H_2_ evolution from HCl, we turned our attention to identifying a photoredox mediator that could function under acidic conditions. We now examine the role of phosphines as photoredox mediators under acidic conditions (p*K*_a_ in CH_3_CN : PPh_3_ = 7.64; pyridine = 12.53).^24^ The photochemical homolysis of P–H bonds of 2° phosphines generates phosphinyl radicals that display sufficient lifetime (∼160 μs) to participate in halogen-atom abstraction from a Ni(ii) halide complex to furnish a reduced Ni intermediate that participates in an H_2_ evolution cycle; the phosphine photoredox mediator is regenerated by HAA from solvent to close the photocycle.^25–27^ The H_2_-evolution cycle may eventually be closed by thermally promoted protolytic H_2_ evolution with HCl.‡Crystallographic data for **2**[ClPPh_3_]: C_36_H_30_Cl_4_P_2_Ni, *M* = 725.05, orthorhombic, *Pbca*, *a* = 17.435(4), *b* = 15.662(3), *c* = 24.139(9), *V* = 6591(2), *Z* = 8, *μ* = 1.036 mm^–1^, *T* = 100(2) K, *R*_1_ = 0.0527, w*R*_2_ = 0.0720 (based on all reflections), GooF = 1.030, reflections measured = 57 380, unique reflections = 5858, *R*_int_ = 0.0741. Crystallographic data for **6**: C_40_H_48_Cl_4_O_4_P_2_Ni, *M* = 855.23, monoclinic, *P*2_1_/*n*, *a* = 10.1261(8), *b* = 23.4579(18), *c* = 17.8192(14), *β* = 106.3550(12)°, *V* = 4061.4(5), *Z* = 4, *μ* = 0.859 mm^–1^, *T* = 100(2) K, *R*_1_ = 0.0788, w*R*_2_ = 0.1328 (based on all reflections), GooF = 1.005, reflections measured = 44 051, unique reflections = 7212, *R*_int_ = 0.0668. Crystallographic data for Cl_2_PPh_3_: C_18_H_15_Cl_2_P, *M* = 333.17, monoclinic, *P*2_1_/*c*, *a* = 13.338(3), *b* = 14.376(3), *c* = 8.7454(17), *β* = 102.53(3)°, *V* = 1637.0(6), *Z* = 4, *μ* = 0.484 mm^–1^, *T* = 100(2) K, *R*_1_ = 0.0330, w*R*_2_ = 0.0710 (based on all reflections), GooF = 1.060, reflections measured = 18 333, unique reflections = 2874, *R*_int_ = 0.0308. Crystallographic data for [ClPPh_3_]OTf: C_19_H_15_ClF_3_O_3_PS, *M* = 446.79, monoclinic, *P*2_1_/*n*, *a* = 11.255(2), *b* = 9.1501(18), *c* = 18.658(4), *β* = 93.04(3)°, *V* = 1918.7(7), *Z* = 4, *μ* = 0.438 mm^–1^, *T* = 100(2) K, *R*_1_ = 0.0425, w*R*_2_ = 0.0737 (based on all reflections), GooF = 1.031, reflections measured = 18 058, unique reflections = 3392, *R*_int_ = 0.0362.


## Experimental

### Materials and methods

All reactions were carried out in an N_2_-filled glovebox. Anhydrous solvents were obtained by filtration through drying columns.^28^ NMR chemical shifts are reported in ppm with the residual solvent resonance as internal standard. UV–vis spectra were recorded at 293 K in quartz cuvettes on a Spectral Instruments 400 series diode array and were blanked against the appropriate solvent. PhICl_2_^29^ and Ni(PPh_3_)_2_(CH_2_ =CH_2_)^30^ were prepared according to reported procedures. NiCl_2_dme (dme = 1,2-dimethoxyethane) and AgOTf (OTf = trifluoromethanesulfonate) were obtained from Strem Chemicals. Cl_2_PPh_3_, prepared by treatment of PPh_3_ with PhICl_2_, displayed spectral features identical to those reported in the literature.^31^ NiCl_2_(PPh_3_)_2_ (**1**), Ni(PPh_3_)_4_ (**5**), tetrabutylammonium chloride (^*n*^Bu_4_NCl), tetraethylammonium chloride (^*n*^Et_4_NCl), and triphenylphosphine (PPh_3_) were obtained from Sigma Aldrich. All chemicals were used without further purification. Elemental analysis was obtained by Complete Analysis Laboratories, Inc., New Jersey. Evolved hydrogen was quantified by gas chromatography using a calibration curve derived from adding HCl to known quantities of NaH; over the relevant concentration range, the gas chromatograph response was linear. This procedure has previously been validated by comparison with Toepler pump combustion analysis.^15^

### Preparation of Ni(ii) trihalide complexes

#### Complex **2**[ClPPh_3_]

A solution of PhICl_2_ (9.1 mg, 3.30 × 10^–5^ mol, 1.00 equiv.) in 1 mL of CH_2_Cl_2_ was added to a solution of NiCl_2_(PPh_3_)_2_ (21.4 mg, 3.30 × 10^–5^ mol, 1.00 equiv.) in 2 mL of CH_2_Cl_2_ to prompt an immediate colour change from a light beige to blue. The solvent was removed *in vacuo*, and the resulting solid was treated with pentane. The pentane was decanted, and the resulting solid was dried *in vacuo* to afford 21.5 mg of title compound (90% yield). ^1^H NMR (600 MHz, CD_3_CN) *δ* (ppm): 7.77 (m, 9H), 7.64 (m, 6H). *μ*_eff_ (CH_3_CN) = 4.20 *μ*_B_. Anal. calcd (found) for C_36_H_30_Cl_4_NiP_2_: C, 59.63 (59.53); H, 4.17 (4.09). Crystals suitable for single-crystal diffraction analysis were obtained from a CH_3_CN solution of the complex layered with Et_2_O.

#### Complex **2**[TBA]

To a suspension of NiCl_2_(dme) (40.0 mg, 1.82 × 10^–4^ mol, 1.00 equiv.) in CH_2_Cl_2_ was added PPh_3_ (47.7 mg, 1.82 × 10^–4^ mol, 1.00 equiv.) and ^*n*^Bu_4_NCl (50.6 mg, 1.82 × 10^–4^ mol, 1.00 equiv.) as a solid. The reaction solution immediately turned from yellow to blue. The reaction mixture was stirred at 23 °C for 1 h. The reaction was concentrated to dryness and the residue was taken up in pentane and Et_2_O, solvent was decanted, and the residue was dried *in vacuo* to afford 119 mg of the title complex as a blue solid (98% yield). ^1^H NMR (600 MHz, CD_3_CN) *δ* (ppm): 3.32 (q, 2H), 1.83 (m, 2H), 1.53 (m, 2H), 1.08 (t, 3H). *μ*_eff_ (CH_3_CN) = 4.02 *μ*_B_. Anal. calcd (found) for C_34_H_51_Cl_3_NNiP: C, 60.97 (60.93); H, 7.68 (7.58); N, 2.09 (2.06). Complex **2**[TEA] was prepared analogously by substitution of ^*n*^Bu_4_NCl with ^*n*^Et_4_NCl in 95% yield; ^1^H NMR (600 MHz, CD_3_CN) *δ* (ppm): 3.48 (q, 2H), 1.45 (t, 3H). *μ*_eff_ (CH_3_CN) = 4.17 *μ*_B_. Anal. calcd (found) for C_26_H_35_Cl_3_NNiP: C, 56.01 (56.14); H, 6.33 (6.26); N, 2.51 (2.67). Crystals suitable for single-crystal diffraction analysis were obtained from a CH_3_CN solution of the complex layered with Et_2_O and unit cell data matched literature reports.^32^

### Preparation of [ClPPh_3_]OTf

To a solution of Cl_2_PPh_3_ (127 mg, 3.81 × 10^–4^ mol, 1.00 equiv.) in CH_2_Cl_2_ was added AgOTf (98.0 mg, 3.81 × 10^–4^ mol, 1.00 equiv.) as a suspension in CH_2_Cl_2_. White solid immediately precipitated when AgOTf was added and the reaction mixture was stirred at 23 °C for 1 h before being filtered through Celite. The filtrate was concentrated *in vacuo* and the residue was taken up in THF and solvent was decanted, and the residue was dried *in vacuo* to afford 157 mg of the title compound as a white solid (92% yield). ^31^P NMR (160 MHz, CD_2_Cl_2_) *δ* (ppm): 66.4; ^19^F NMR (275 MHz, CD_2_Cl_2_) *δ* (ppm): –78.9. The spectral data is consistent with that reported for ClPPh_3_·AlCl_4_.^33^ Crystals suitable for single-crystal diffraction analysis were obtained from a CH_2_Cl_2_ solution layered with Et_2_O.

### Preparation of Ni(ii) tetrachloride complex [NiCl_4_][Et_4_N]_2_

A solution of ^*n*^Et_4_Cl (30.2 mg, 1.82 × 10^–4^ mol, 1.00 equiv.) in 2 mL of CH_2_Cl_2_ was added to a solution of NiCl_2_(dme) (40.0 mg, 1.82 × 10^–4^ mol, 1.00 equiv.) in 2 mL of CH_2_Cl_2_ to prompt an immediate colour change from yellow to green. After stirring at 23 °C for 0.5 h, the reaction mixture was concentrated to dryness and the residue was taken up in pentane and solvent was decanted, and the residue was dried *in vacuo* to afford 77.2 mg of the title compound as a green solid (92% yield). Crystals suitable for single-crystal diffraction analysis were obtained from a CH_3_CN solution layered with Et_2_O and unit cell data matched literature reports.^34^

### Preparation of Ni(i) complexes

#### Complex **3**

To a scintillation vial was added Ni(cod)_2_ (58.0 mg, 2.10 × 10^–4^ mol, 1.00 equiv.) and NiCl_2_(dme) (46.0 mg, 2.10 × 10^–4^ mol, 1.00 equiv.) as solids, followed by 3 mL of PhCH_3_. To this solution was added PPh_3_ (330 mg, 1.26 × 10^–3^ mol, 6.00 equiv.) dissolved in 2 mL PhCH_3_ and the reaction mixture was stirred at 23 °C for 12 h before being filtered through Celite. The filtrate was concentrated *in vacuo* to a volume of 1.5 mL, layered with hexanes, and cooled to –30 °C to afford yellow crystalline solid (80% yield). ^1^H NMR (600 MHz, THF-*d*_8_) *δ* (ppm): 9.51 (br, s, 20H), 5.28 (br, s, 15H), 4.20 (br, s, 10H). *μ*_eff_ (CH_3_CN) = 1.74 *μ*_B_. Crystals suitable for single-crystal diffraction analysis were obtained from a PhCH_3_ solution of the complex layered with *n*-hexane and collected unit cell data matched literature reports.^35^

#### Complex **4**

To a scintillation vial was added Ni(PPh_3_)_2_(CH_2_ =CH_2_) (20.0 mg, 3.30 × 10^–5^ mol, 1.00 equiv.) and NiCl_2_(PPh_3_)_2_ (21.4 mg, 3.30 × 10^–5^ mol, 1.00 equiv.) as solids followed by 4 mL of Et_2_O. The reaction mixture was stirred at 23 °C for 0.5 h, during which time a yellow precipitate was observed. The mixture was concentrated to dryness and the residue was taken up in pentane and dried *in vacuo* to afford 18.4 mg of the title complex as a yellow solid (90% yield).^36^

## Results and discussion

**Fig. 1:**
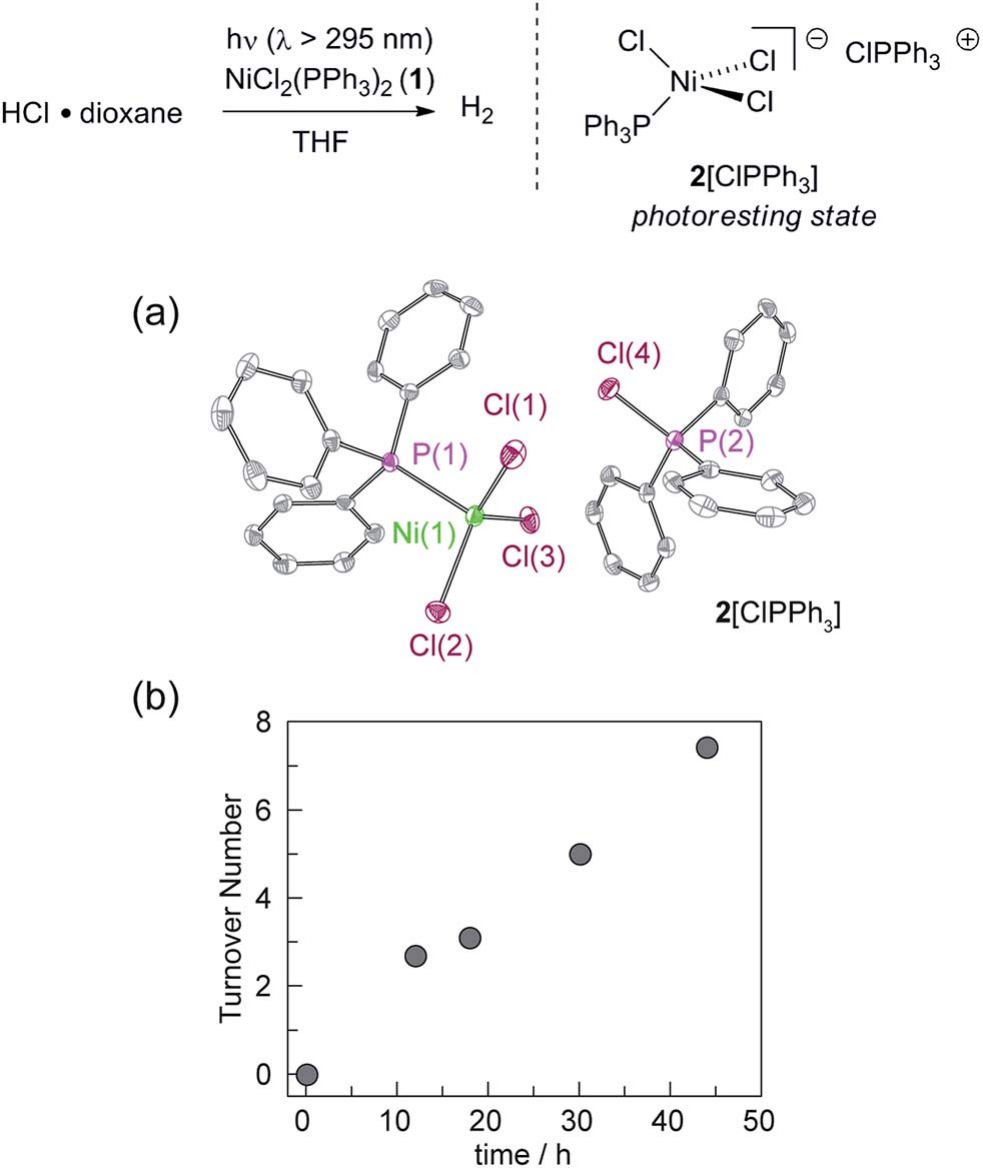
Photolysis of NiCl_2_(PPh_3_)_2_ in THF (*λ* > 295 nm) in the presence of 15 equiv. of HCl affords H_2_ as well as **2**[ClPPh_3_]. (a) Thermal ellipsoid plot of **2**[ClPPh_3_] drawn at the 50% probability level. The hydrogen atoms are omitted for clarity. (b) Time-dependent turnover number (TON) of H_2_ produced by a 4.5 mM THF solution of NiCl_2_(PPh_3_)_2_ in the presence of 15 equiv. HCl (*λ* > 295 nm).

We targeted phosphines as potential photoredox mediators for a tandem photoredox/transition metal catalysed H_2_-evolution photocycle from HCl based on their demonstrated ability to serve as photochemical H-atom donors.^27^ To evaluate the viability of the proposed phosphine-mediated photoredox approach for H_2_ evolution, we photolyzed Ni(ii) complex NiCl_2_(PPh_3_)_2_ (**1**) in THF (*λ* > 295 nm) in the presence of 1.0 equiv. PPh_3_ and 15 equiv. of HCl. The light beige reaction solution turned pale blue upon photolysis. Analysis of the headspace by gas chromatography (GC) confirmed H_2_ as the exclusive gaseous product under these conditions; integration of the chromatogram and comparison to a H_2_ calibration curve generated from the reaction of NaH with HCl revealed that 3.1 turnovers had been achieved in 18 h. NiCl_2_(PPh_3_)_2_ participates in ligand dissociation equilibria to release PPh_3_ (*vide infra*),^37^ and thus H_2_-evolving photocatalysis was also observed in the absence of exogenous PPh_3_. Evaluation of the amount of H_2_ evolved as a function of time (Fig. 1), showed that in the presence of excess HCl, H_2_ evolution continues and 9 turnovers were achieved after 44 h with no signs of deactivation.

During the photolysis of NiCl_2_(PPh_3_)_2_, the blue-coloured solution that initially develops persists throughout subsequent H_2_ evolution and based on UV-vis measurements, appears to represent the photoresting state of the catalyst system. The pale blue photoproduct was identified as Ni(ii) trihalide complex **2**[ClPPh_3_] by single-crystal X-ray diffraction analysis.‡ In addition, comparison of the *in situ* UV-vis spectrum obtained during H_2_-evolving catalysis with a spectrum obtained from an authentic sample of **2**[ClPPh_3_], prepared by treatment of NiCl_2_(PPh_3_)_2_ with 1.0 equiv. of PhICl_2_ (Fig. S15†), confirmed the identity of the catalyst resting state. Independently isolated complex **2**[ClPPh_3_] is chemically competent at H_2_ generation under above photoreaction conditions.

Catalyst resting state **2**[ClPPh_3_] is constituted of a Ni trichloride anion and a phosphonium cation. In order to establish the roles of both the Ni complex and phosphine in the observed H_2_ evolution reaction, **2**[TBA] and phosphonium cation [ClPPh_3_]OTf were independently prepared. As summarized in Table 1, Ni(ii) complex **2**[TBA] showed a similar activity toward HCl with 5.0 TON in 18 h. Complex **2**[TBA] also participates in a minor equilibrium with free PPh_3_ (*vide infra*) and thus both catalyst and photoredox mediator are present when **2**[TBA] is employed as the photocatalyst. In contrast, phosphonium salt [ClPPh_3_]OTf does not produce H_2_ under the same reaction conditions. Additionally, neither PPh_3_ or [NiCl_4_][TEA]_2_ is a competent H_2_-evolution catalyst, confirming the necessity of both Ni complex and phosphine for productive H_2_ evolution chemistry.

**Table 1:** TON of H_2_ measured in the headspace upon photolysis of designated compounds in the presence of 15 equiv. HCl in THF for 18 h

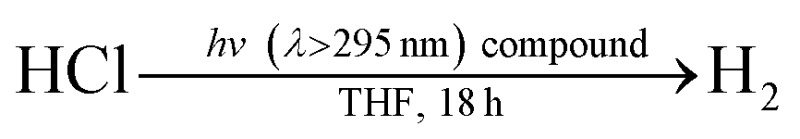	
Compound	TON
**2**[ClPPh_3_]	2.0
PPh_3_	0
[NiCl_4_][Et_4_N]_2_	0
**2**[TBA]	5.0
[ClPPh_3_]OTf	0

**Fig. 2:**
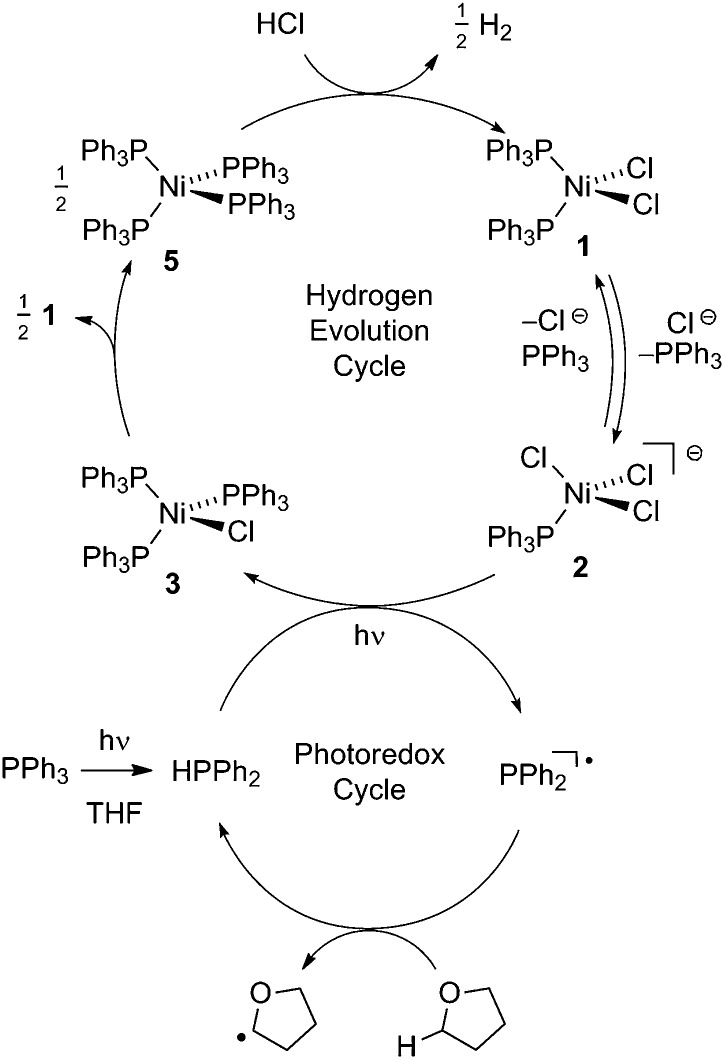
Proposed tandem catalytic cycles for H_2_-generation with Ni-based H_2_-evolution catalysts and phosphine-based photoredox mediators.

Fig. 2 illustrates a tandem catalytic cycle that accounts for the photogeneration of H_2_ from HCl catalyzed by the Ni phosphine complexes and phosphine photoredox mediators. Diphenyl phosphine is initially formed by photochemical cleavage of the P–C bond and H-atom abstraction from solvent.^27^ Photochemical cleavage of the P–H bond in HPPh_2_ generates an H-atom equivalent and a diphenylphosphinyl radical.^27^ The H-atom participates in halogen-atom abstraction with Ni(ii) resting state **2** to generate a Ni(i) intermediate while the accompanying diphenylphosphinyl radical participates in C–H abstraction with solvent to regenerate the diphenylphosphine and close the photoredox cycle. Ni(i) intermediate **3** undergoes disproportionation to afford NiCl_2_(PPh_3_)_2_ (**1**) and Ni(PPh_3_)_4_ (**5**). Protonolysis of Ni(0) complex **5** affords H_2_ and regenerates Ni(ii) dihalide **1**, thus closing the hydrogen evolution cycle. The number of phosphine ligands bound to intermediates in the tandem cycle illustrated in Fig. 2 is unknown. Isolated complexes **2**[ClPPh_3_], **3**, **4** and **5**, which display 1–4 phosphine ligands per Ni, are all competent catalysts for H_2_ evolution and proceed with the same photoresting state (**2**[ClPPh_3_]), demonstrating ligand dissociation equilibria^37^ are established during catalysis (Fig. S16†).

In order to probe the contention that photogenerated diphenylphosphinyl radicals could mediate the reduction of a Ni–Cl bond from the Ni(ii) trihalide complex, we carried out time-resolved photochemical experiments. On the picosecond timescale, a transient absorption (TA) difference spectrum obtained by laser flash photolysis (*λ*_exc_ = 310 nm, THF solutions) of **2**[TBA] exhibits a spectral growth centred at 506 nm with a lifetime of ∼700 ps (Fig. S18†). An identical spectral feature was observed during laser flash photolysis of PPh_3_ solutions. This feature was assigned to be that of the singlet excited state of PPh_3_, and the observation of this signal in the TA spectrum of **2**[TBA] supports the presence of a minor equilibrium between **2**[TBA] and free PPh_3_.^38^ Additional support for this ligand dissociation equilibrium is the observation that both **2**[TBA] and PPh_3_ both show an emission band centred at 500 nm with a 900 ps lifetime (Fig. S17†), which is well-matched to reported PPh_3_ photophysics.^39^ The similar emission lifetimes for both **2**[TBA] and PPh_3_ excludes dynamic quenching of the excited ^1^PPh_3_* species by the Ni complex and suggests that the relatively low steady-state emission intensity observed for **2**[TBA] is due only to a low equilibrium concentration of PPh_3_.

**Fig. 3:**
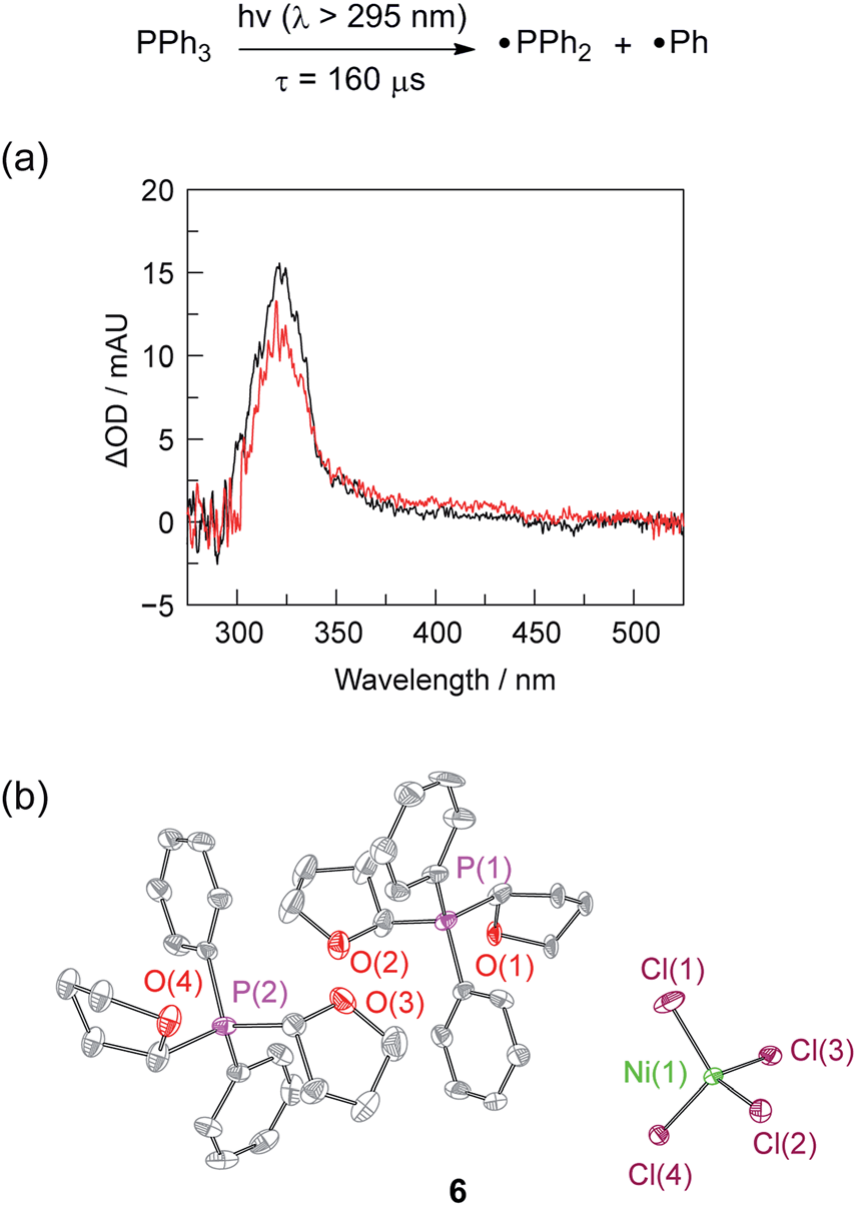
(a) Nanosecond transient absorption (TA) spectroscopy of Ni complex **2**[TEA] (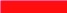, red) and PPh_3_ (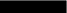, black) is consistent with formation of diphenylphosphinyl radical. TA spectrum obtained by laser flash photolysis (310 nm pump) of a 1 : 1 THF : CH_3_CN solution recorded at a 1 μs delay. (b) Thermal ellipsoid of **6** drawn at the 50% probability level. The hydrogen atoms are omitted for clarity.

The photochemistry of **2** and PPh_3_ were also examined at longer time scales by nanosecond flash photolysis (Fig. 3). Flash photolysis of either **2**[TEA] (red spectrum, Fig. 3) or PPh_3_ (black spectrum, Fig. 3) leads to the observation of TA signals that are ascribed to diphenylphosphinyl radical.^27^ The lifetime of the diphenylphosphinyl radical derived from PPh_3_ with and without the presence of **2**[TBA] in solution is the same, consistent with no direct reaction between Ni(ii) complex and diphenylphosphinyl radical (Fig. S19†). Substantial phosphine consumption is not required for H_2_ evolution because the diphenylphosphine generated during catalysis is a competent photoredox carrier. Nanosecond-resolved TA spectra, collected by laser flash photolysis of diphenylphosphine in THF, display the spectral features of diphenylphosphinyl radical (Fig. S20†), confirming that phosphine mediators can be catalytic.^27,40^

That the photogenerated diphenylphosphinyl radical engages in HAA with THF to produce diphenylphosphine is confirmed by the isolation of complex **6**, as a photo-byproduct of the photolysis of Ni complex **2**[TEA]. Complex **6** features two C-bound tetrahydrofuranyl ligands on a phosphorus centre and could be derived from reaction of photogenerated diphenylphosphinyl radicals with furanyl radicals derived from HAA chemistry. Additionally we observed (GC-MS) octahydro-2,2′-bifuran (homocoupled THF) as a photochemical byproduct. While HAA from THF by diphenylphosphinyl radicals is endothermic (16 kcal mol^–1^ uphill based on P–H and C–H bond dissociation energies),^41^ irreversible subsequent reactions, such as radical coupling to afford homocoupled THF sequester reactive radical intermediates.^40^

The rate for HAA from solvent by photogenerated diphenylphosphinyl is strongly correlated with solvent C–H bond energies.^27,42^ We therefore anticipated that efficiency of the total catalytic process would be dictated by the turnover frequency of the photoredox mediator, which depends on the C–H BDEs of H-atom donor. The turnover frequency (TOF) of hydrogen generation from HCl with Ni complex **2** strongly depends on the solvent we employed, showing a positive correlation to the BDE of solvents: 0.34 h^–1^ TOF in THF, 0.05 h^–1^ TOF in CH_3_CN, and 0.02 h^–1^ TOF in C_6_H_6_ (92, 96, and 112 kcal mol^–1^ of C–H BDEs respectively) (see Fig. S21† for details).^43–45^

To probe the potential fate of potential Ni(i) intermediates during catalysis, we examined the chemistry of isolated Ni(i) complexes. Ni(i) complex **3** can be isolated from the comproportionation reaction of NiCl_2_(PPh)_2_ with Ni(PPh_3_)_4_. Based on the *E*°(Ni^II^/Ni^I^) and *E*°(Ni^I^/Ni^0^) measured by cyclic voltammetry in THF (Fig. S22†), comproportionation is thermodynamically favored. In contrast, in the presence of exogenous chloride ion, added as tetrabutylammonium chloride, disproportionation of Ni(i) complex **3** to Ni(ii) complex **1** and Ni(0) complex **5** is observed, as determined by both ^31^P NMR and electronic absorption spectroscopy (Fig. S8 and S14,† respectively). During H_2_ evolution photocatalysis, chloride is present in large excess (68 mM) with respect to potential Ni(i) intermediates.
1

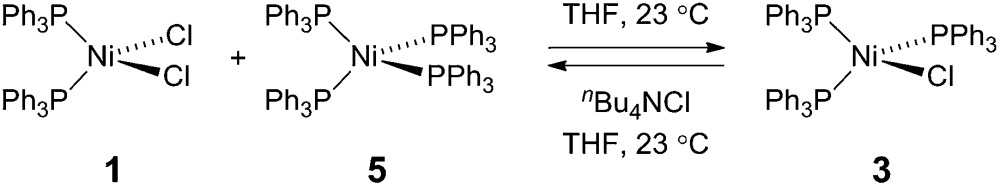




**Fig. 4:**
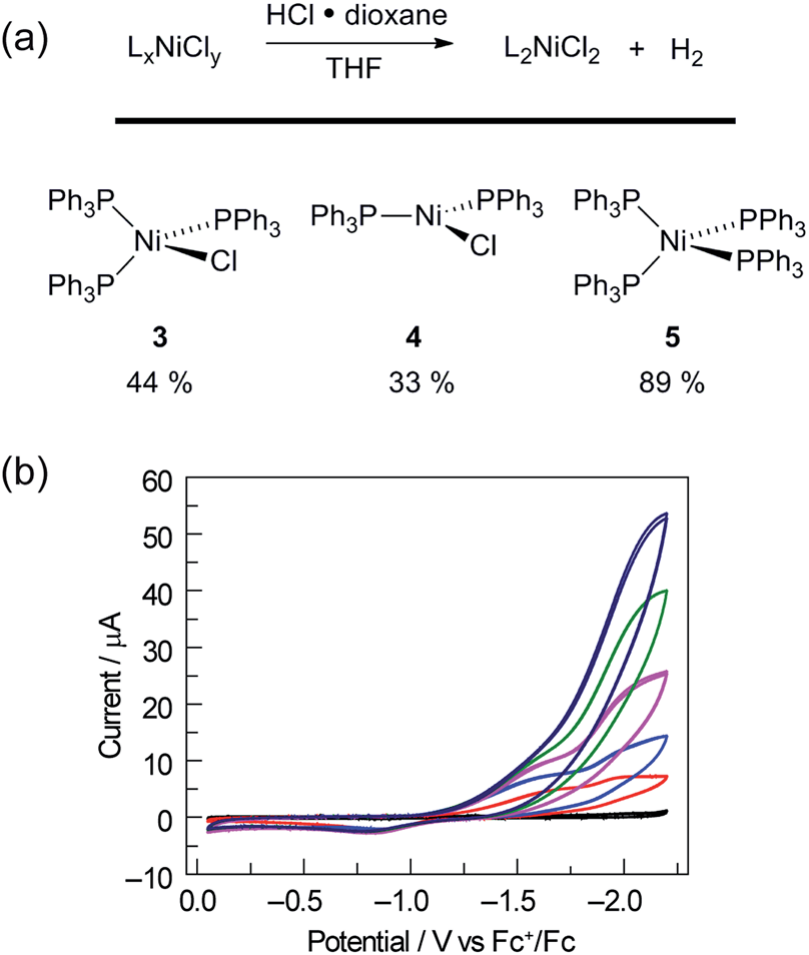
(a) Protonation of Ni(i) and Ni(0) complexes afforded Ni(ii)chloride as well as H_2_. (b) Electrochemical response of electrolyte background (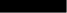, black), 1 mM Ni complex **2**[TEA] (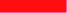, red) to addition of HCl 1.0 equiv. (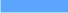, blue), 5.0 equiv. (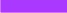, pink), 9.0 equiv. (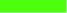, green), 13.0 equiv. (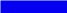, dark blue) in CH_3_CN (0.1 M NBu_4_PF_6_; scan rate, 100 mV s^–1^). Glassy carbon working electrode, Ag/AgNO_3_ reference, and Pt wire counter electrode.

To assess whether the initially produced Ni(i) complex (**3**) or the Ni(0) complex (**5**) generated by disproportionation are active for H_2_ production, we examined the stoichiometric H_2_-evolution reaction chemistry of Ni(i) and Ni(0) complexes with HCl. The results of these experiments are summarized in Fig. 4. Treatment of Ni complexes **3**, **4**, and **5** with 15 equiv. HCl in THF generates H_2_ in 44, 33, and 89% yields, respectively, along with Ni(ii) complex **1**, NiCl_2_(PPh_3_)_2_. To gain insight into whether H_2_ evolution proceeds by protonation of Ni(i) or Ni(0), generated by disproportionation reactions, electrochemical H_2_ evolution was examined using Ni(ii) trihalide complex **2**[TEA]. As illustrated in Fig. 4, Ni complex **2**[TEA] exhibits two electrochemically irreversible waves for the Ni^II/I^ and Ni^I/0^ couples at –1.62 and –1.95 V *vs.* Fc^+^/Fc, respectively. In the presence of excess HCl (p*K*_a_ = 8.9 in CH_3_CN)^46^ these two peaks exhibit catalytic cathodic waves. The dominant CV features of the Ni^I/0^ wave in the presence of excess HCl are consistent with Ni^0^ being involved in the H_2_ generating steps in our photocatalysis.

## Conclusions

As is common for first-row transition metal complexes, nickel halide complexes typically exhibit very short excited state lifetimes. Direct photoactivation of M–X bonds using the molecular excited states of these complexes has proven challenging owing to their short lifetimes. To circumvent the limitations imposed by short excited state lifetimes, we have developed a tandem photoredox/transition metal catalysis approach to H_2_ evolution in which the chromophore and the H_2_-evolution catalyst are localized on different molecules. Using diaryl phosphines as photoredox mediators, we have demonstrated that relatively non-basic phosphines are capable of acting as photoredox mediators under acidic conditions. Robust photocatalytic systems have been developed by combining phosphine photoredox mediators and Ni phosphine H_2_-evolution catalysts. Time-resolved spectroscopy has revealed that phosphines serve as photochemical H-atom donors and activate the M–X bonds of Ni(ii) halide complexes *via* halogen-atom abstraction. The H_2_-evolution catalytic cycle is closed by sequential disproportionation of Ni(i) to afford Ni(0) and Ni(ii) and protolytic H_2_ evolution from the Ni(0) intermediate. The described photoredox strategy is attractive in that independent optimization of photoredox mediator and H_2_-evolution catalyst provides multiple handles for system optimization.

## References

[cit1] Esswein A. J., Nocera D. G. (2007). Chem. Rev..

[cit2] Nocera D. G. (2009). Inorg. Chem..

[cit3] Heyduk A. F., Nocera D. G. (2001). Science.

[cit4] Esswein A. J., Veige A. S., Nocera D. G. (2005). J. Am. Chem. Soc..

[cit5] Elgrishi N., Teets T. S., Chambers M. B., Nocera D. G. (2012). Chem. Commun..

[cit6] Cook T. R., Surendranath Y., Nocera D. G. (2009). J. Am. Chem. Soc..

[cit7] Teets T. S., Nocera D. G. (2009). J. Am. Chem. Soc..

[cit8] Lin T.-P., Gabbaï F. P. (2012). J. Am. Chem. Soc..

[cit9] Yang H., Gabbaï F. P. (2014). J. Am. Chem. Soc..

[cit10] Van Zyl W. E., López-de-Luzuriaga J. M., Fackler Jr J. P., Staples R. J. (2001). Can. J. Chem..

[cit11] Fackler Jr J. P. (2002). Inorg. Chem..

[cit12] Ovens J. S., Leznoff D. B. (2011). Dalton Trans..

[cit13] Perera T. A., Masjedi M., Sharp P. R. (2014). Inorg. Chem..

[cit14] Karikachery A. R., Lee H. B., Masjedi M., Ross A., Moody M. A., Cai X., Chui M., Hoff C. D., Sharp P. R. (2013). Inorg. Chem..

[cit15] Powers D. C., Chambers M. B., Teets T. S., Elgrishi N., Anderson B. L., Nocera D. G. (2013). Chem. Sci..

[cit16] Carrera E. I., McCormick T. M., Kapp M. J., Lough A. J., Seferos D. S. (2013). Inorg. Chem..

[cit17] For a recent counter-example, see: Powers D. C. Hwang S. J. Zheng S.-L. Nocera D. G., Inorg. Chem., 2014, 53 , 9122 . 25137532 10.1021/ic501136m

[cit18] Lee C. H., Lutterman D. A., Nocera D. G. (2013). Dalton Trans..

[cit19] Tereniak S. J., Marlier E. E., Lu C. C. (2012). Dalton Trans..

[cit20] Juban E. A., Smeigh A. L., Monat J. E., McCusker J. K. (2010). Coord. Chem. Rev..

[cit21] Creutz C., Chou M., Netzel T. L., Okumura M., Sutin N. (1980). J. Am. Chem. Soc..

[cit22] Lee C. H., Cook T. R., Nocera D. G. (2011). Inorg. Chem..

[cit23] Powers D. C., Anderson B. L., Nocera D. G. (2013). J. Am. Chem. Soc..

[cit24] Haav K., Saame J., Kütt A., Leito I. (2012). Eur. J. Org. Chem..

[cit25] Sakaguchi Y., Hayashi H. (2004). J. Phys. Chem. A.

[cit26] Versace D.-L., Bastida J. C., Lorenzini C., Cachet-Vivier C., Renard E., Langlois V., Malval J.-P., Fouassier J.-P., Lalevée J. (2013). Macromolecules.

[cit27] Wong S. K., Sytnyk W., Wan J. K. S. (1971). Can. J. Chem..

[cit28] Pangborn A. B., Giardello M. A., Grubbs R. H., Rosen R. K., Timmers F. J. (1996). Organometallics.

[cit29] Zhao X.-F., Zhang C. (2007). Synthesis.

[cit30] Schramm K. D., Ibers J. A. (1980). Inorg. Chem..

[cit31] Yano T., Hoshino M., Kuroboshi M., Tanaka H. (2010). Synlett.

[cit32] Smith M. C., Davies S. C., Hughes D. L., Evans D. J. (2001). Acta Crystallogr., Sect. E: Struct. Rep. Online.

[cit33] Schmidpeter A., Lochschmidt S. (1990). Inorg. Synth..

[cit34] Stucky G. D., Folkers J. B., Kistenmacher T. J. (1967). Acta Crystallogr..

[cit35] Ellis D. D., Spek A. L. (2000). Acta Crystallogr., Sect. C: Cryst. Struct. Commun..

[cit36] Norman N. C., Orpen A. G., Quayle M. J., Whittell G. R. (2002). Acta Crystallogr., Sect. C: Cryst. Struct. Commun..

[cit37] Bontempelli G., Magno F., Nobili M. D., Schiavon G. (1980). J. Chem. Soc., Dalton Trans..

[cit38] Sakaguchi Y., Hayashi H. (1995). Chem. Phys. Lett..

[cit39] Maini L., Braga D., Mazzeo P. P., Ventura B. (2012). Dalton Trans..

[cit40] Kaufman M. L., Griffin C. L. (1965). Tetrahedron Lett..

[cit41] Waterman R. (2008). Curr. Org. Chem..

[cit42] Roth J. P., Lovell S., Mayer J. M. (2000). J. Am. Chem. Soc..

[cit43] Laarhoven L. J. J., Mulder P., Wayner D. D. M. (1999). Acc. Chem. Res..

[cit44] Cherkasov A., Jonsson M. (2000). J. Chem. Inf. Comput. Sci..

[cit45] van Scheppingen W., Dorrestijn E., Arends I., Mulder P. (1997). J. Phys. Chem. A.

[cit46] Fourmond V., Jacques P.-A., Fontecave M., Artero V. (2010). Inorg. Chem..

