# Supplementary material for: Tandem redox mediator/Ni(ii) trihalide complex photocycle for hydrogen evolution from HCl
Source: Chem Sci. 2014 Oct 8;6(2):917–22. doi: 10.1039/c4sc02357a (PMC5811117; doi:10.1039/c4sc02357a)
Supplement: Supplementary file 1 [file SC-006-C4SC02357A-s001.pdf]

Electronic Supplementary Material (ESI) for Chemical Science.
This journal is © The Royal Society of Chemistry 2014

Supporting Information

Tandem Redox Mediator/Ni(II) Trihalide Complex Photocycle for Hydrogen Evolution from HCl

Seung Jun Hwang,[a] David C. Powers,[a] Andrew G. Maher[a,b] and Daniel G. Nocera[a,*]

[a]*Department of Chemistry and Chemical Biology, Harvard University, 12 Oxford Street, Cambridge, MA 02138–2902*

[b] *Department of Chemistry, Massachusetts Institute of Technology, 77 Massachusetts Avenue, Cambridge, MA 02139-4307*

Email: dnocera@fas.harvard.edu

Table of Contents *Page*

A. Physical Methods S3

B. Reaction of $NiCl(PPh_3)_3$ with $^nBu_4NCl$ S5

Reaction of $NiCl(PPh_3)_3$ with nBu_4NCl S5

Charaterization of $Ni(PPh_3)_2(CH_2{=}CH_2)$ S5

C. NMR Data S6

D. X-Ray Data Analysis S14

Structural Data for Ni(II) Trichloride **2**[$ClPPh_3$] S14

Structural Data for Ni(II) Tetrachloride **6** ([$NiCl_4$][$PPh_2(THF)_2$]$_2$) S16

Structural Data for Dichlorophosphine, PPh_3Cl_2 .... S18

Structural Data for Monochlorophosphine, [$ClPPh_3$]OTf S20

E. UV-vis Absorption Spectroscopy S22

F. Fluoresence Emission Spectroscopy S25

G. Picosecond Transient Absorption Spectroscopy S26

H. Nanosecond Transient Absorption Spectroscopy S27

I. Turnover frequency (TOF) in different solvents S28

I. Electrochemical Data S29

A. Physical Methods

NMR spectra were recorded at the Harvard University Department of Chemistry and Chemical Biology NMR facility on a Varian Unity / Inova 600 spectrometer operating at 600 MHz for ^{1}H acquisitions or a or a Varian Mercury 400 spectrometer operating at 375 MHz and 160 MHz for ^{19}F and ^{31}P acquisitions, respectively. NMR chemical shifts are reported in ppm with the residual solvent resonance as internal standard. ^{31}P NMR chemical shifts were referenced to an external 85% H_3PO_4 standard. UV-vis spectra were recorded at 293 K in quartz cuvettes on a Spectral Instruments 400 series diode array and were blanked against the appropriate solvent. Steady-state emission spectra were obtained using a PTI QM 4 Fluorometer, with a 150 W Xe-arc lamp used for excitation (set to 310 nm) and a Hamamatsu R928 photomultiplier tub used for detection. Solution magnetic moments were determined using the Evans method in THF and measured using ^{19}F NMR (hexafluorobenzene added); diamagnetic corrections were estimated from Pascal constants.[1] Steady-state photochemical reactions were performed using a 1000 W high–pressure Hg/Xe arc lamp (Oriel) and the beam was passed through a water–jacketed filter holder containing the appropriate long–pass filter, an iris, and a collimating lens. Structures were collected on a Bruker three-circle platform goniometer equipped with an Apex II CCD and an Oxford cryostream cooling device at 100 K. Radiation was supplied from either a graphite fine focus sealed tube Mo Kα (0.71073 Å) source. Crystals were mounted on a glass fibre using Paratone N oil. Data were collected as a series of φ and/or ω scans. Data were integrated using SAINT and scaled with either a numerical or multi-scan absorption correction using SADABS. The structures were solved by intrinsic phasing methods using SHELXS-97 and refined against F^2 on all data by full matrix least squares with SHELXL-97. All non-hydrogen atoms were refined anisotropically. Hydrogen atoms were placed at idealized positions and refined using a riding model.

Picosecond transient absorption (TA) experiments were performed using a previously reported home-built Ti:sapphire laser system.[2] The excitation wavelength was set to 310 nm with the power kept between 10-20 μJ/pulse at the sample. The continuum for the probe pulses was generated by focusing an 800 nm beam on a CaF_2 substrate. The reported experiments used a 500 nm blaze grating (300 grooves/mm) and the entrance slit for the monochromator was set to 0.3 mm. The transient absorption spectra reported are averages of 4 replicates of 500 four-spectrum sequences. To remove artifacts in the baseline, spectra were corrected by subtracting an average of 3 spectra taken at negative delays, i.e. time points at which the probe pulse arrives at the sample before the pump pulse. Single-wavelength kinetics traces were obtained by averaging 5 nm spectral windows about the

---

1 G. A. Bain and J. F. Berry, *J. Chem. Ed.* 2008, **85**, 532–536.

2 D. C. Powers, B. L. Anderson and D. G. Nocera, *J. Am. Chem. Soc.* 2013, **135**, 18876.

wavelength of interest for each time point. The reported time constants were calculated using a least-squares fit of the data to a monoexponential decay on the OriginPro 8.5 data analysis software. Samples for picosecond TA experiments were prepared using THF in 2.0 mm path-length quartz high-vacuum spectroscopy cells and freeze-pump-thawed for 3 cycles using high-vacuum (1.0×10^{-5} torr). Nanosecond TA experiments were performed using a modified version of a previously-reported home-built Nd:YAG laser system.[3] In the modified setup, the previously-used Triax 320 spectrometer has been replaced by a Horiba iHR320 spectrometer. The output of the Xe-arc lamp was set to 2.0 ms pulses with 30 A current. The reported experiments used a 250 nm blaze grating (300 grooves/mm). For the full-spectrum TA acquisitions, the entrance slit for the monochromator was set to 0.32 mm and the gate time for the CCD was 55 ns. For the single wavelength kinetics TA acquisitions (centered at 320 nm), the entrance and exit slits were set to 0.16 mm, and a 1.0 kV bias was applied to the photomultiplier tube detector. Error bars for the lifetime data correspond to the standard error of the monoexponential fits. The pump beam (300 nm) was generated from the frequency-doubled 600 nm signal of a Spectra-Physics Quanta-Ray MOPO-700 with FDO–970 option pumped with the 355 nm light from the aforementioned laser. The power of the pump beam was set to 2.4 mJ/pulse. The full TA spectra reported are averages of 200 four-spectrum sequences, and the single wavelength decays are averages of 500 acquisitions. THF or THF:CH_3CN (1:1) solutions of complex 2 and triphenylphosphine were prepared in 20-mL vials sealed with rubber septa in an N_2-filled glovebox. Solutions were flowed through a 3-mm diameter, 1-cm path length flow cell (Starna, type 585.2) using a peristaltic pump and positive argon pressure. Time-resolved emission data was collected using a Hamamatsu C4334 Streak Scope camera, with the 310 nm excitation pulses provided by the Ti:sapphire laser system described above, set to a 1 kHz repetition rate. A 5 ns time window was used, and 5000 exposures were captured for each sample. Samples for both steady-state and time-resolved emission experiments were prepared using THF in 1.0 cm quartz high-vacuum spectroscopy cells in a N_2-atmosphere glovebox.

[3] P. G. Holder, A. A. Pizano, B. L. Anderson, J. Stubbe and D. G. Nocera, *J. Am. Chem. Soc.* 2012, **134**, 1172.

B. Synthesis and Characterization

Reaction of $NiCl(PPh_3)_3$ with nBu_4NCl

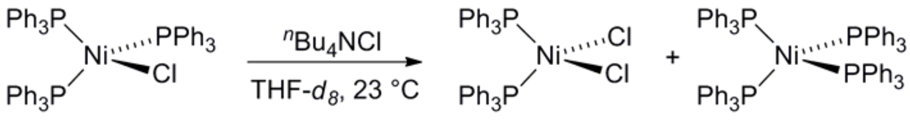

3 1 5

To a solution of $NiCl(PPh_3)_3$ (3) (14.1 mg, 1.60×10^{-5} mol, 1.00 equiv) in THF-d_8 was added $^nBu_4NCl$ (4.5 mg, 1.6×10^{-4} mol, 1.0 equiv) as a solid at 23 °C. The yellow reaction solution assumed a brown orange color immediately and $Ni(PPh_3)_4$ (5) is observed by ^{31}P NMR (reproduced in Figure S8). Neither $NiCl(PPh_3)_3$ (3) or $NiCl_2(PPh_3)_2$ (1) display ^{31}P NMR signals.

Charaterization of Ni$(PPh_3)_2(CH_2{=}CH_2)$

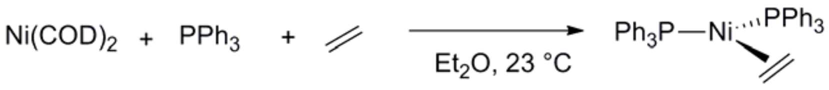

$Ni(PPh_3)_2(CH_2{=}CH_2)$ were prepared according to reported procedures described above,[4] but NMR data have not been reported in literature ^1H NMR (600 MHz, C_6D_6) δ (ppm): 7.51 (m, 13H), 6.97 (m, 20H), 2.63(s, 4H). (See ^1H NMR data in Figure S6).

---

4 K. D. Schramm and J. A. Ibers, *Inorg. Chem.* 1980, **19**, 2441.

C. NMR Data

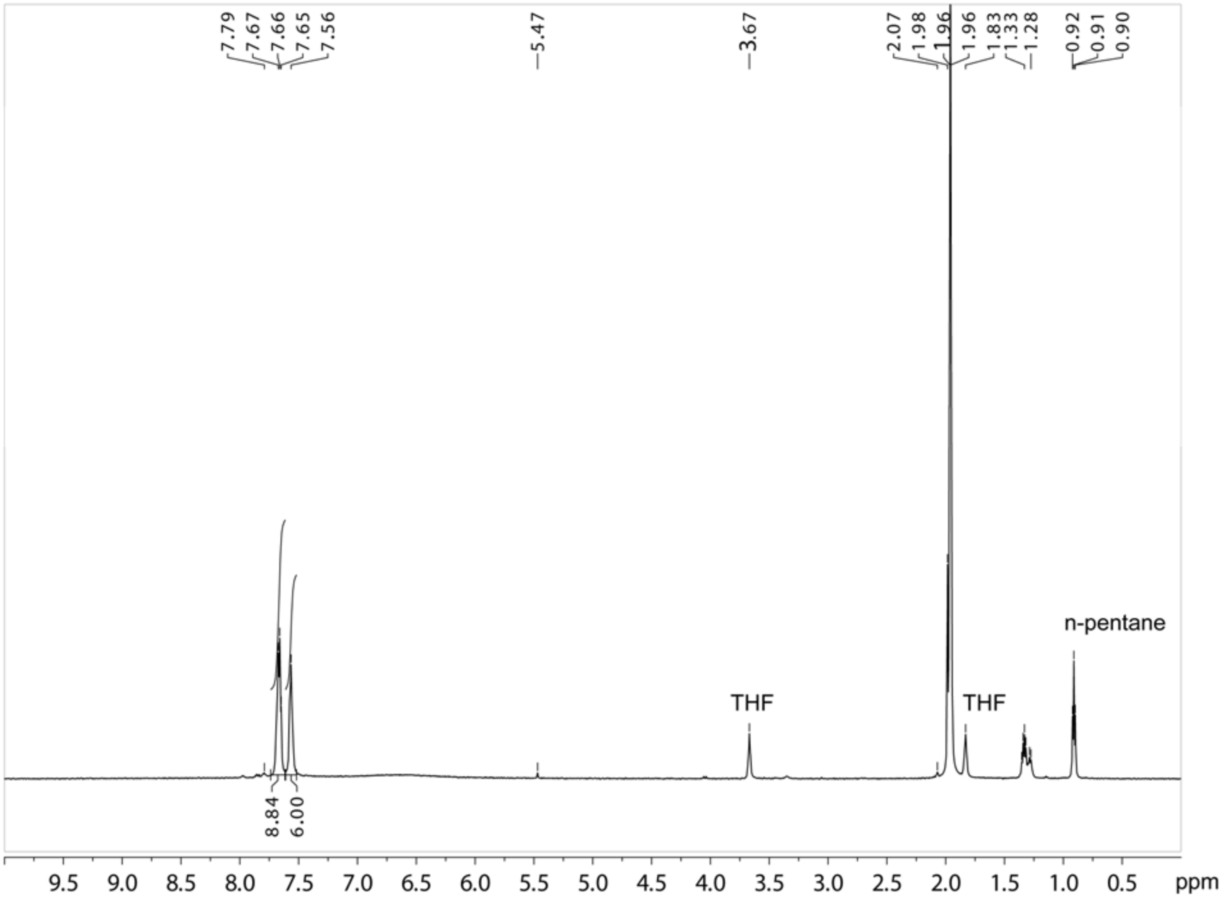

Figure S1. ^1H NMR spectrum of **2**[$ClPPh_3$] recorded in CD_3CN at 23 °C.

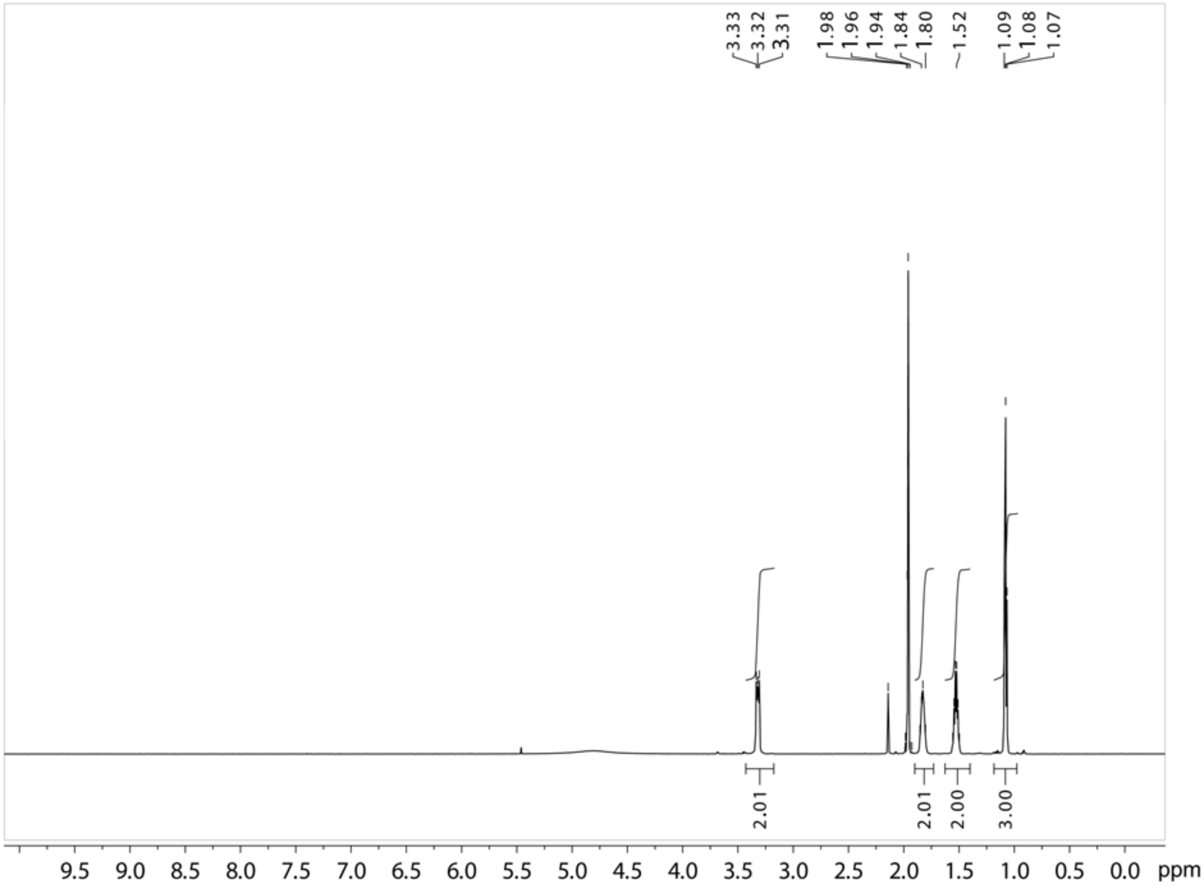

Figure S2. ^{1}H NMR spectrum of **2**[TBA] recorded in CD_3CN at 23 °C.

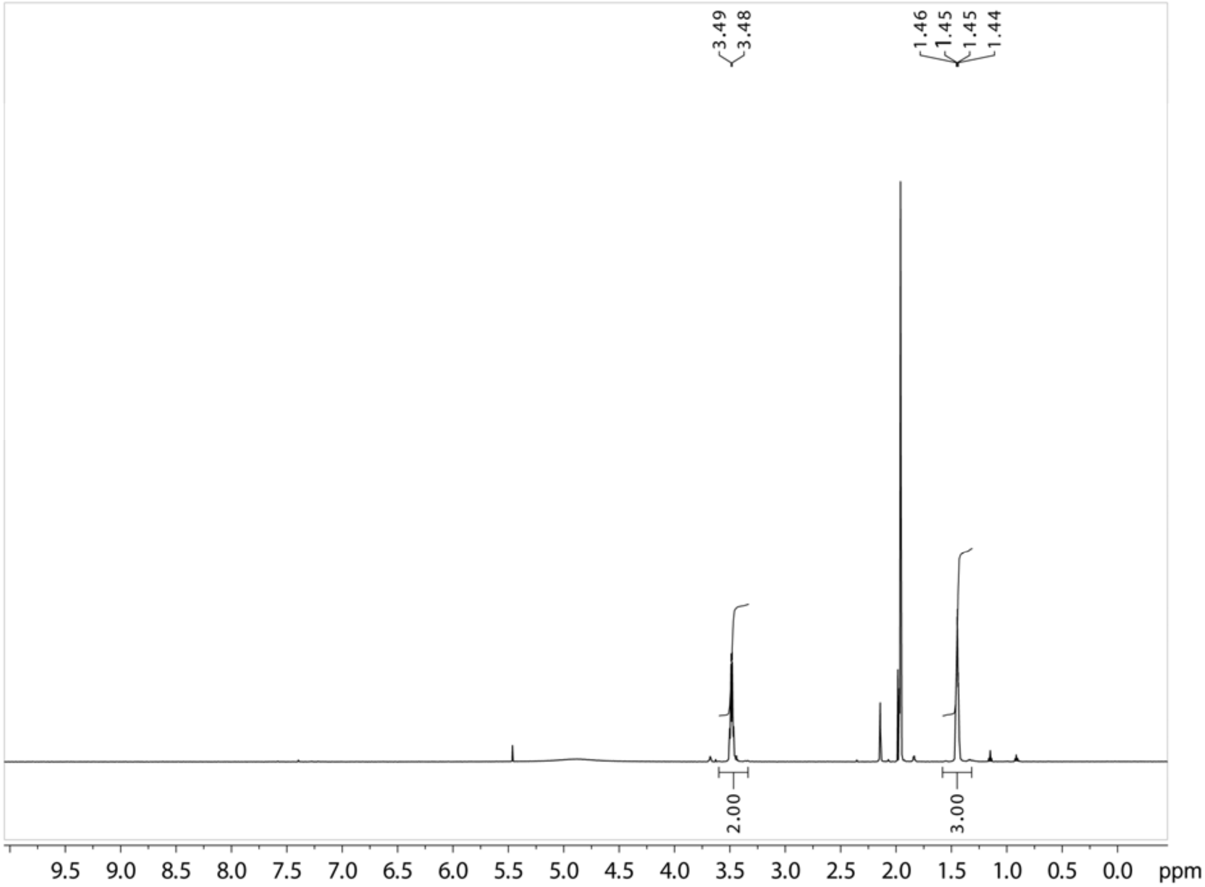

Figure S3. ^{1}H NMR spectrum of **2**[TEA] recorded in CD_3CN at 23 °C.

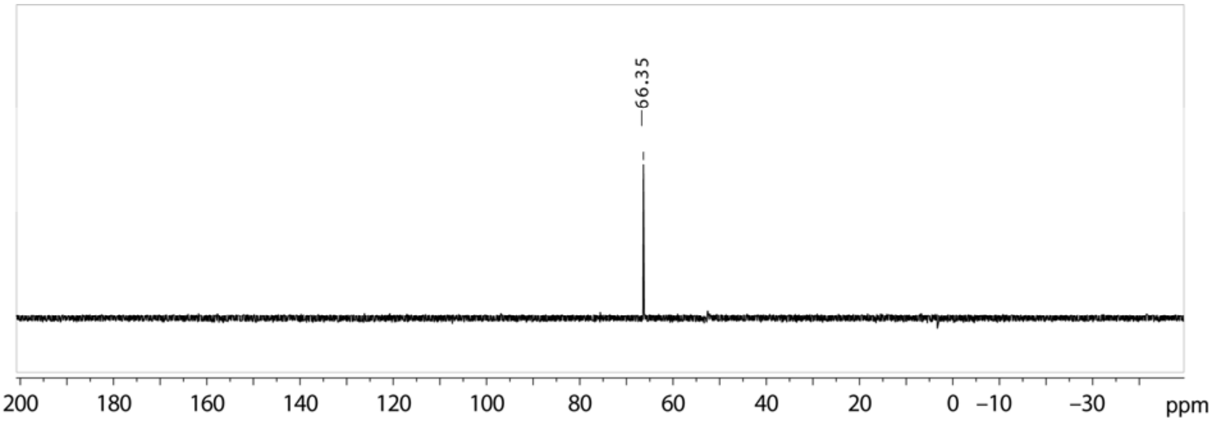

Figure S4. ^{31}P NMR spectrum of $[ClPPh_3]OTf$ recorded in CD_2Cl_2 at 23 °C.

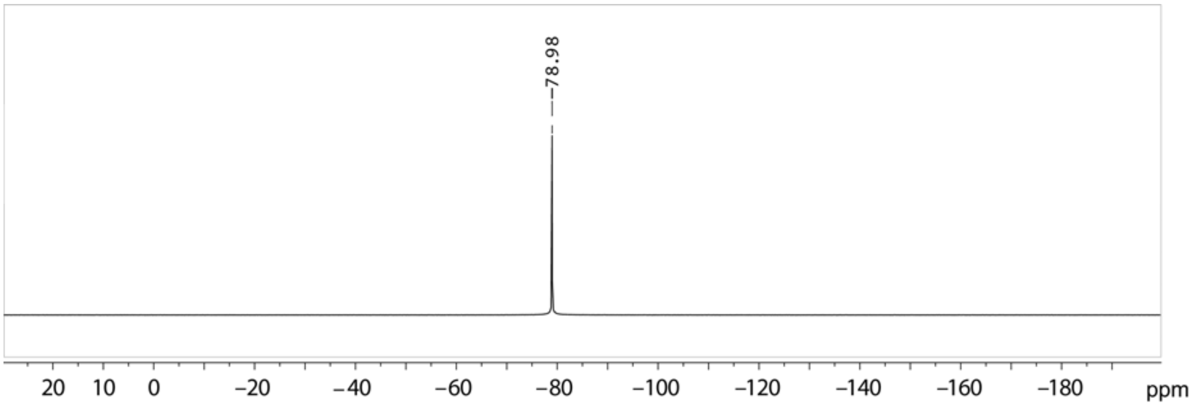

Figure S5. ^{19}F NMR spectrum of $[ClPPh_3]OTf$ recorded in CD_2Cl_2 at 23 °C.

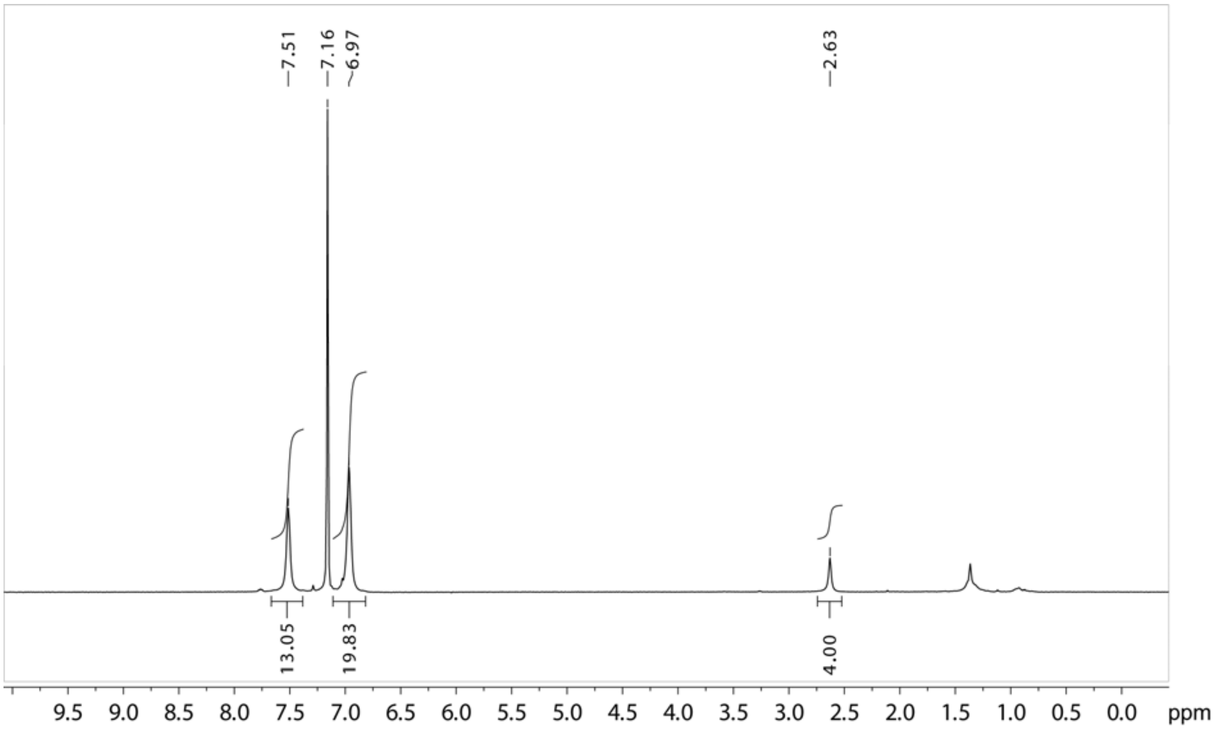

Figure S6. ^1H NMR spectrum of $Ni(PPh_3)_2(CH_2{=}CH_2)$ recorded in C_6D_6 at 23 °C.

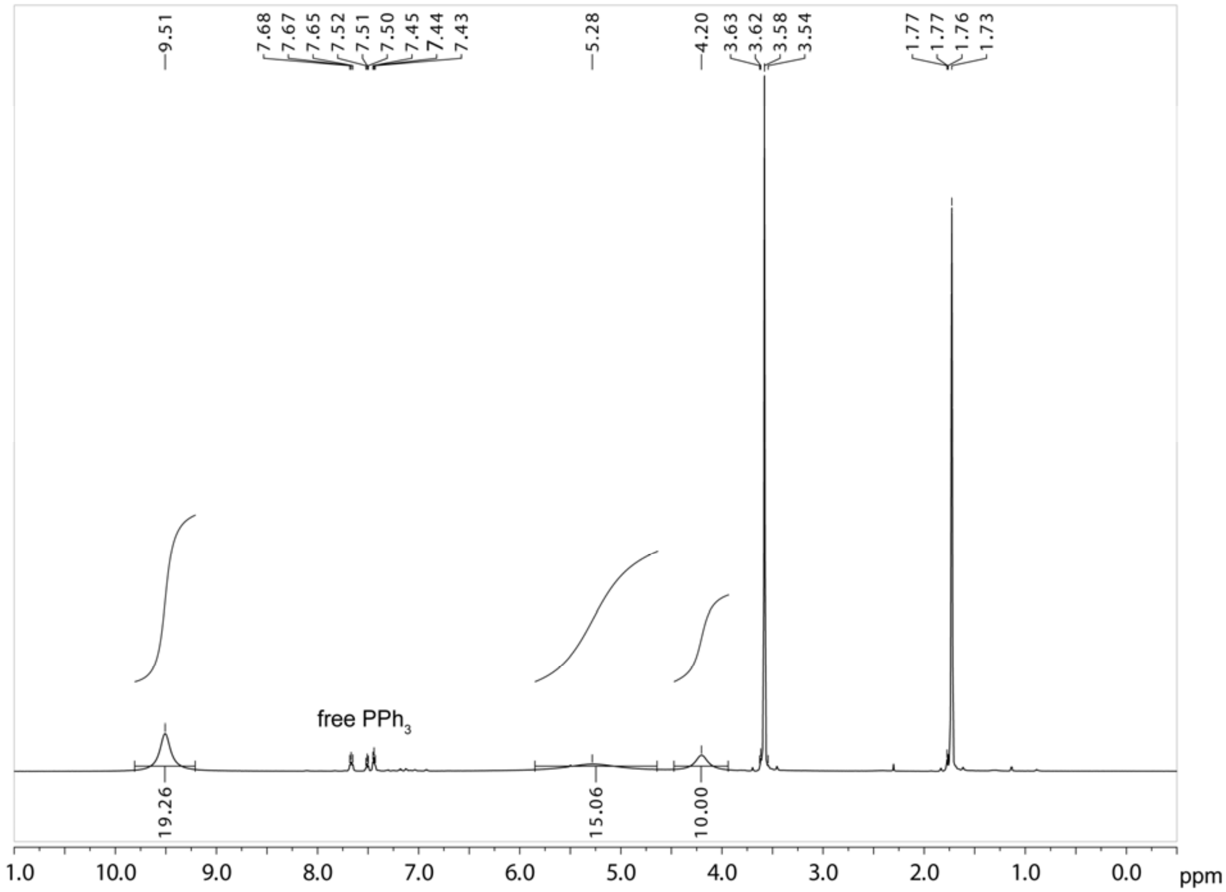

Figure S7. ^{1}H NMR spectrum of $NiCl(PPh_3)_3$ (**3**) recorded in THF-d_8 at 23 °C.

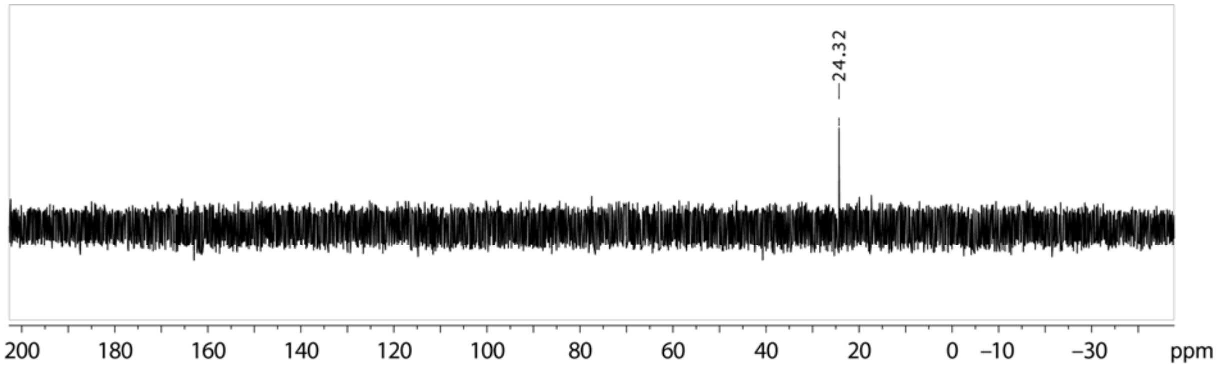

Figure S8. ^{31}P NMR spectrum of $NiCl(PPh_3)_3$ (**3**) with 1 equiv of $^nBu_4NCl$ recorded in THF-d_8 at 23 °C. $NiCl(PPh_3)_3$ (**3**) and $NiCl_2(PPh_3)_2$ (**1**) do not display ^{31}P NMR signals.

D. X-Ray Data Analysis

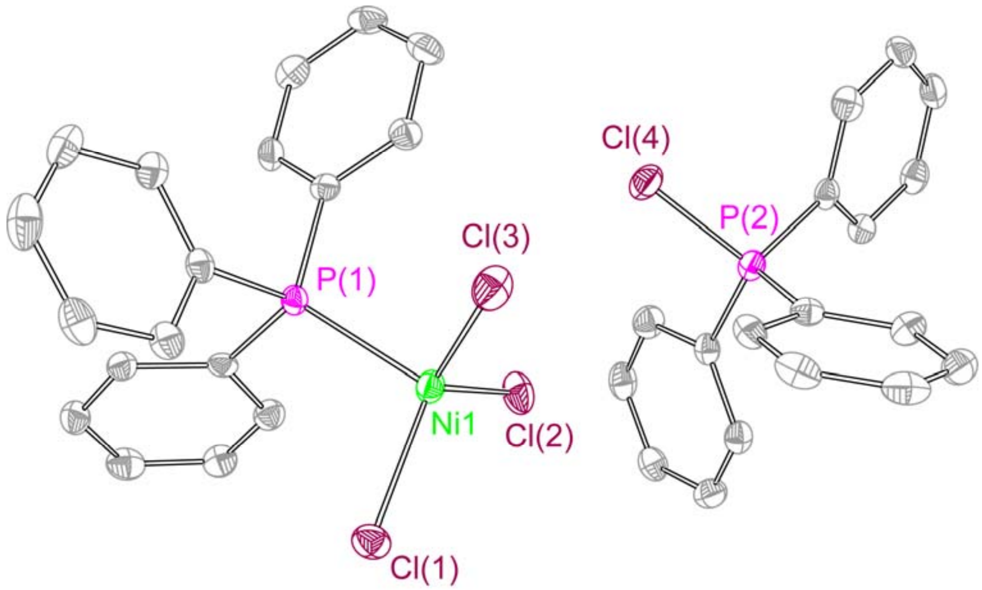

Figure S9. Thermal ellipsoid plot of **2**[$ClPPh_3$] drawn at the 50% probability level. H-atoms are omitted for clarity.

Table S1. X-ray experimental details for $NiCl_3(PPh_3)ClPPh_3$ (**2**[$ClPPh_3$], CCDC 992218)

Crystal Data	
Chemical formula	$C_{36}H_{30}Cl_4NiP_2$
Fw, g/mol	725.05
Crystal system, space group	Orthorhombic, *Pbca*
Temperature (K)	100(2)
a, *b*, *c* (Å)	17.435(4), 15.662(3), 24.139(5)
α, β, γ (°)	90, 90, 90
V (Å^3)	6591(2)
Z	8
Radiation type	Mo *K*α
μ (mm^{-1})	1.036
Crystal size (mm)	0.08 × 0.07 × 0.06
Data collection	
Diffractometer	Bruker *APEX*-II CCD
Absorption correction	Multi-scan, *SADABS*
T_{min}, T_{max}	0.9180, 0.9424
No. of measured, independent and observed [$I > 2\sigma(I)$] reflections	57380, 5858, 4551
R_{int}	0.0741
$(\sin \theta/\lambda)_{\max}$ (Å^{-1})	0.610
Refinement	
$R[F^2 > 2\sigma(F^2)]$, $wR(F^2)$, S	0.0327, 0.0649, 1.03
No. of reflections	5858
No. of parameters	388
No. of restraints	0
H-atom treatment	H atoms treated by a mixture of independent and constrained refinement
$Dr_{\max}$, $Dr_{\min}$ (e Å^{-3})	0.407, –0.265

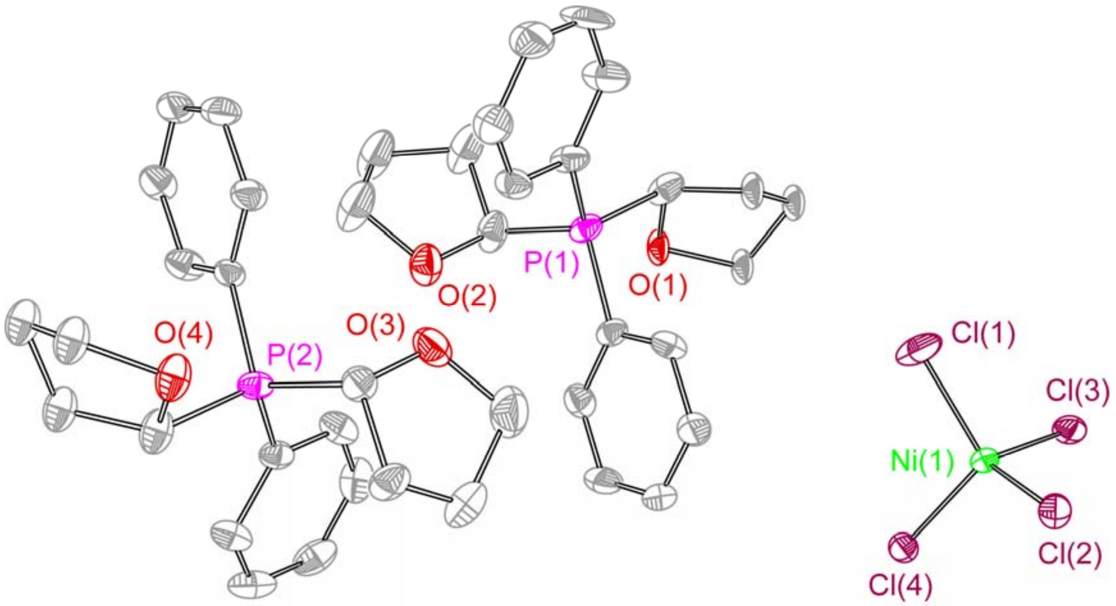

Figure S10. Thermal ellipsoid plot of $[NiCl_4][PPh_2(THF)_2]_2$ (**6**) drawn at the 50% probability level. H-atoms omitted for clarity.

Table S2. X-ray experimental details for $[NiCl_4][PPh_2(THF)_2]_2$ (**6**, CCDC 992221)

Crystal Data	
Chemical formula	$C_{40}H_{48}Cl_4NiO_4P_2$
Fw, g/mol	855.23
Crystal system, space group	Monoclinic, *P2(1)/n*
Temperature (K)	100(2)
a, *b*, *c* (Å)	10.1261(8), 23.4579(18), 17.8192(14)
α, β, γ (°)	90, 106.3550(12), 90
V (Å^3)	4061.4(5)
Z	4
Radiation type	Mo *K*α
μ (mm^{-1})	0.859
Crystal size (mm)	0.58 × 0.36 × 0.24
Data collection	
Diffractometer	Bruker *APEX*-II CCD
Absorption correction	Multi-scan, *SADABS*
T_{min}, T_{max}	0.6370, 0.8191
No. of measured, independent and observed [$I > 2\sigma(I)$] reflections	44051, 7212, 5695
R_{int}	0.0668
$(\sin \theta/\lambda)_{\mathrm{max}}$ (Å^{-1})	0.610
Refinement	
$R[F^2 > 2\sigma(F^2)]$, $wR(F^2)$, S	0.0593, 0.1236, 1.016
No. of reflections	7212
No. of parameters	448
No. of restraints	94
H-atom treatment	H atoms treated by a mixture of independent and constrained refinement
$\mathrm{Dr}_{\mathrm{max}}$, $\mathrm{Dr}_{\mathrm{min}}$ (e Å^{-3})	0.914, –0.883

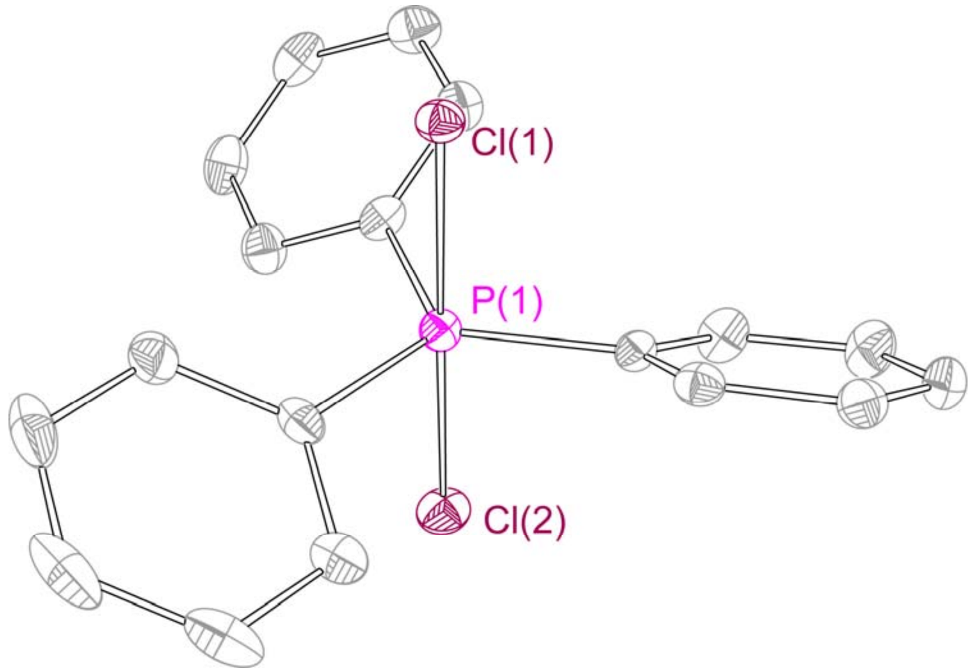

Figure S11. Thermal ellipsoid plot of PPh_3Cl_2 drawn at the 50% probability level. H-atoms omitted for clarity.

Table S3. X-ray experimental details for PPh_3Cl_2 (CCDC 992219)

Crystal Data	
Chemical formula	$C_{18}H_{15}Cl_2P$
Fw, g/mol	333.17
Crystal system, space group	Monoclinic, *P2(1)/c*
Temperature (K)	100(2)
a, *b*, *c* (Å)	13.338(3), 14.376(3), 8.7454(17)
α, *β*, *γ* (°)	90, 102.53(3), 90
V (Å^3)	1637.0(6)
Z	4
Radiation type	Mo *K*α
μ (mm^{-1})	0.484
Crystal size (mm)	0.45 × 0.26 × 0.16
Data collection	
Diffractometer	Bruker *APEX*-II CCD
Absorption correction	Multi-scan, *SADABS*
T_{min}, T_{max}	0.8111, 0.9248
No. of measured, independent and observed [$I > 2\sigma(I)$] reflections	18333, 2874, 2527
R_{int}	0.0308
$(\sin \theta/\lambda)_{\max}$ (Å^{-1})	0.610
Refinement	
$R[F^2 > 2\sigma(F^2)]$, $wR(F^2)$, S	0.0272, 0.0677, 1.060
No. of reflections	2874
No. of parameters	190
No. of restraints	0
H-atom treatment	H atoms treated by a mixture of independent and constrained refinement
$Dr_{\max}$, $Dr_{\min}$ (e Å^{-3})	0.593, –0.301

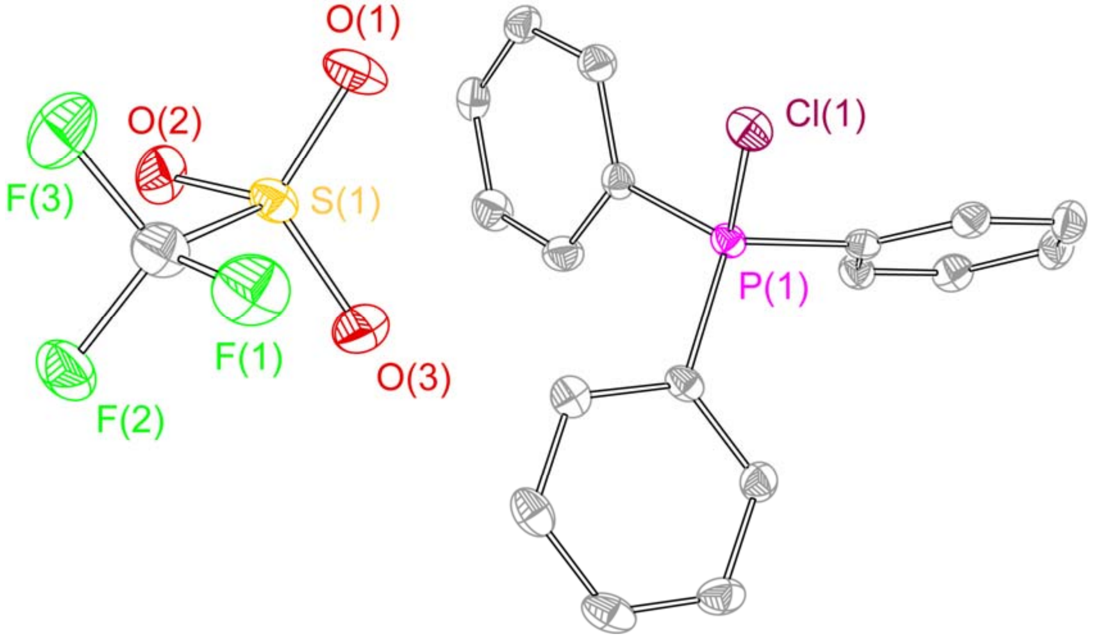

Figure S12. Thermal ellipsoid plot of $[ClPPh_3]OTf$ drawn at the 50% probability level. H-atoms omitted for clarity.

Table S4. X-ray experimental details for [ClPPh$_3$]OTf (CCDC 992220)

Crystal Data	
Chemical formula	$C_{19}H_{15}ClF_3O_3PS$
Fw, g/mol	446.79
Crystal system, space group	Monoclinic, *P2(1)/n*
Temperature (K)	100(2)
a, *b*, *c* (Å)	11.255(2), 9.1501(18), 18.658(4)
α, β, γ (°)	90, 93.04(3), 90
V (Å^3)	1918.7(7)
Z	4
Radiation type	Mo *K*α
μ (mm^{-1})	0.438
Crystal size (mm)	0.14 × 0.09 × 0.08
Data collection	
Diffractometer	Bruker *APEX*-II CCD
Absorption correction	Multi-scan, *SADABS*
T_{min}, T_{max}	0.9417, 0.9642
No. of measured, independent and observed [$I > 2\sigma(I)$] reflections	18058, 3392, 2859
R_{int}	0.0362
$(\sin \theta/\lambda)_{\mathrm{max}}$ (Å^{-1})	0.610
Refinement	
$R[F^2 > 2\sigma(F^2)]$, $wR(F^2)$, S	0.0323, 0.0697, 1.031
No. of reflections	3392
No. of parameters	308
No. of restraints	0
H-atom treatment	H atoms treated by a mixture of independent and constrained refinement
Dr_{max}, Dr_{min} (e Å^{-3})	0.341, –0.283

E. UV-vis Electronic Absorption Spectroscopy

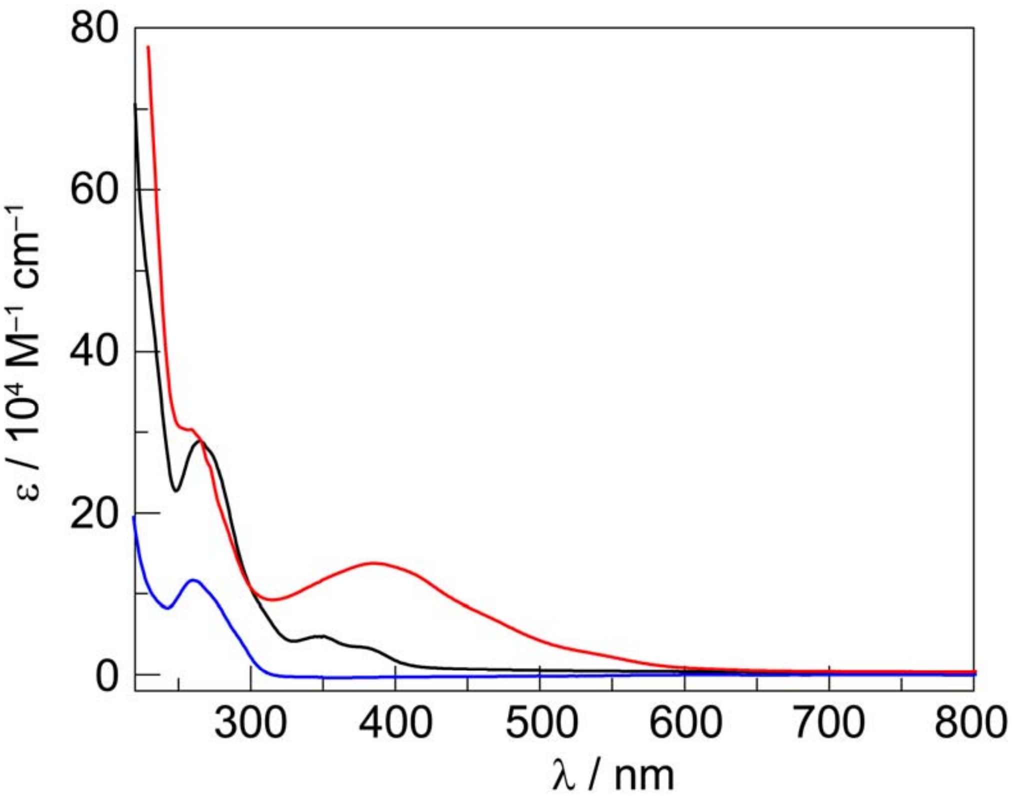

Figure S13. Extinction spectra of Ni complexes $NiCl(PPh_3)_3$ (**3**) (▬, black), $Ni(PPh_3)_4$ (**5**) (▬, red), and **2**[TBA] (▬, blue).

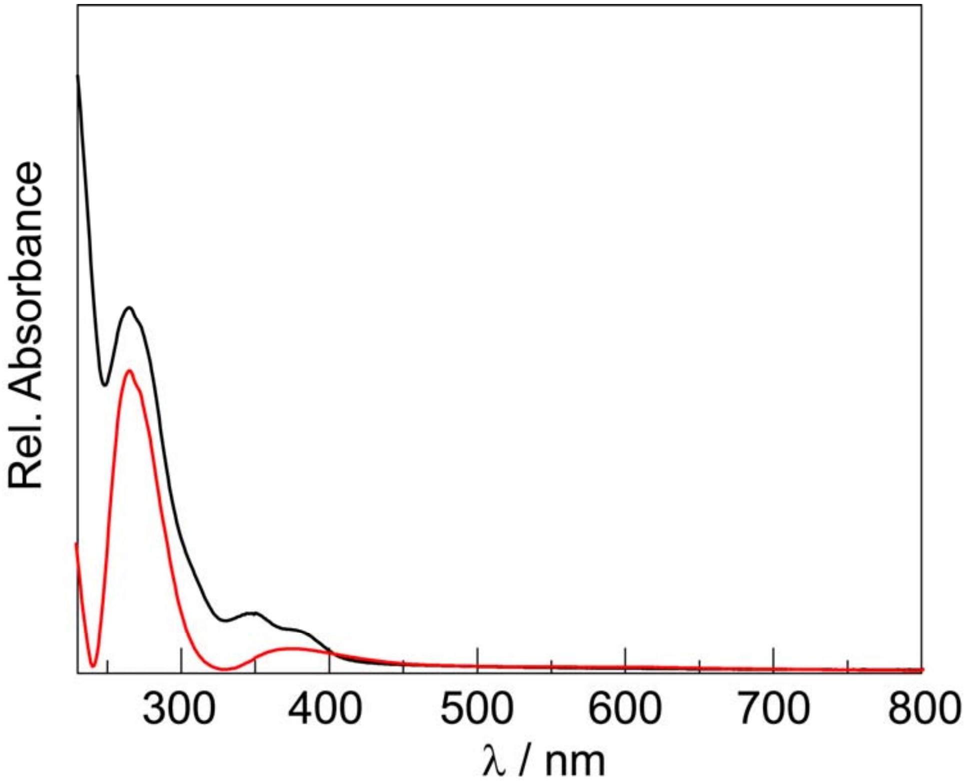

Figure S14. UV-vis spectrum of $NiCl(PPh_3)_3$ (**3**) (▬, black) and $NiCl(PPh)_3$ (**3**) with 1 equiv $^nBu_4Cl$ (▬, red).

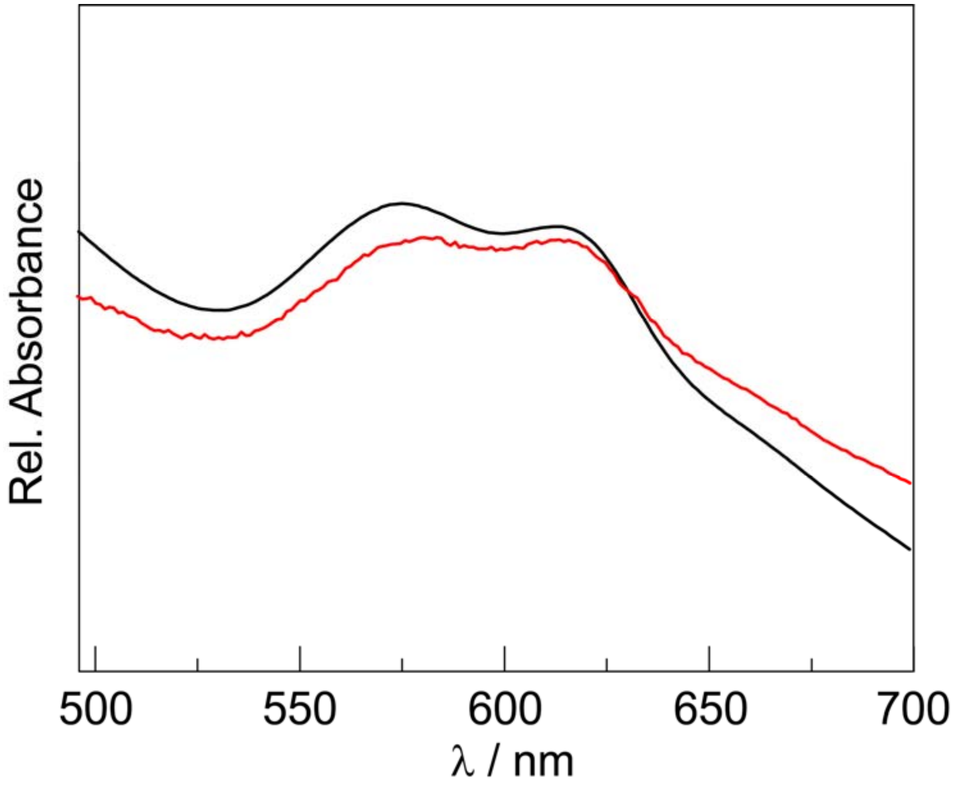

Figure S15. UV-vis spectrum of the photolysis of $NiCl_2(PPh_3)_2$ (**1**) in THF (λ > 295 nm) in the presence of 15 equiv of HCl for 17 h (▬, black), and authentic sample of **2**[$ClPPh_3$] (▬, red) prepared by treatment of $NiCl_2(PPh_3)_2$ with 1.0 equiv of $PhICl_2$.

F. Fluorescence Emission Spectroscopy

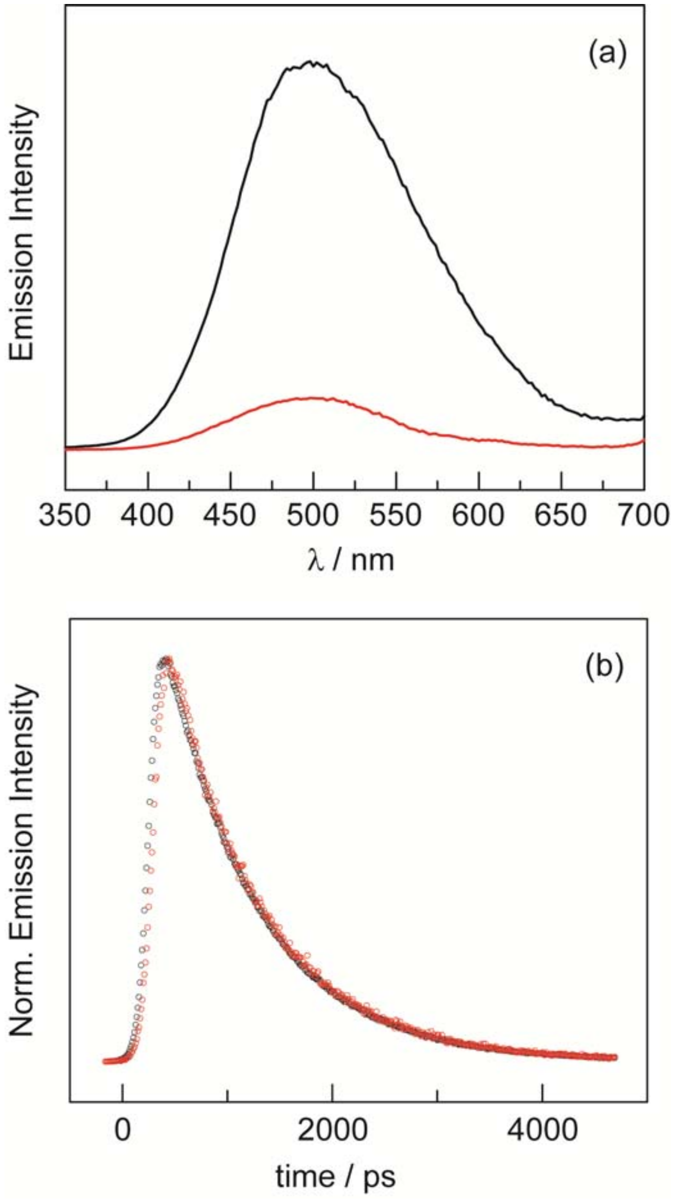

Figure S16. (a) Steady-state emission spectra of **2**[TBA] (▬, red) and PPh_3 (▬, black) in THF. (b) Emission decays of **2**[TBA] (▬, red) and PPh_3 (▬, black) centered at 500 nm.

G. Picosecond Transient Absorption Spectroscopy

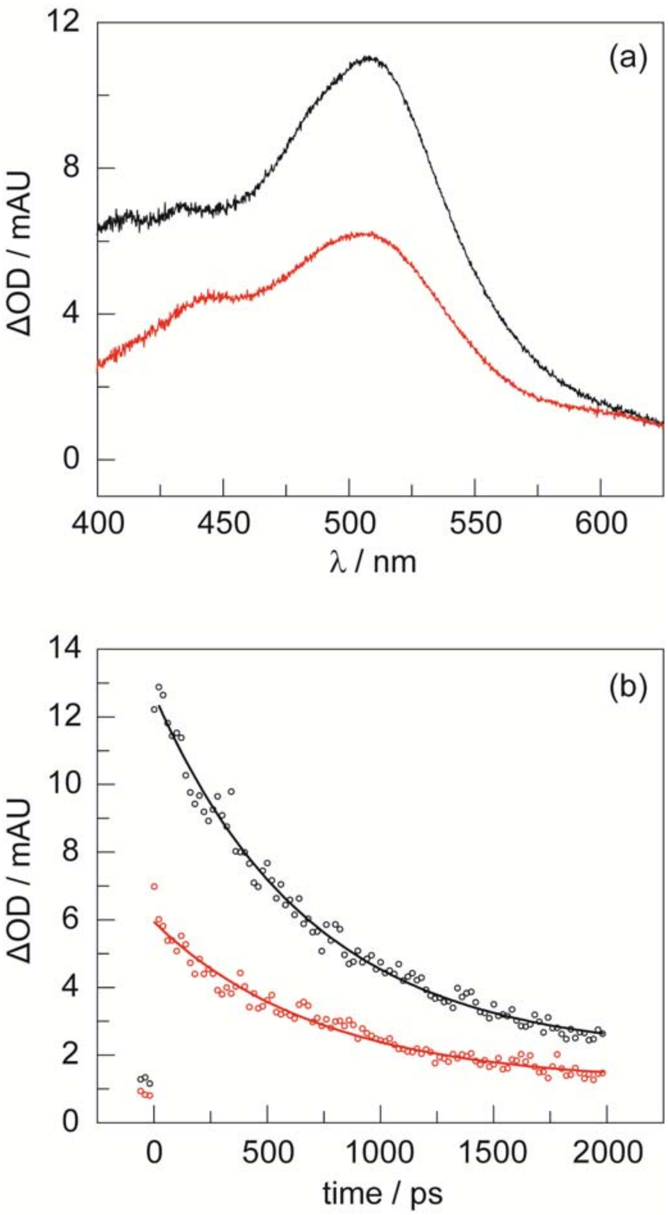

Figure S17. (a) Transient absorption spectra obtained by flash laser photolysis (310 nm pump) of Ni complex **2**[TBA] (▬, red), and PPh_3 (▬, black) in THF at a 2 ps delay. (b) Single wavelength kinetic traces of Ni complex **2**[TBA] (▬, red), and PPh_3 (▬, black) in THF pumped at 310 nm, centered about 506 nm.

H. Nanosecond Transient Absorption Spectroscopy

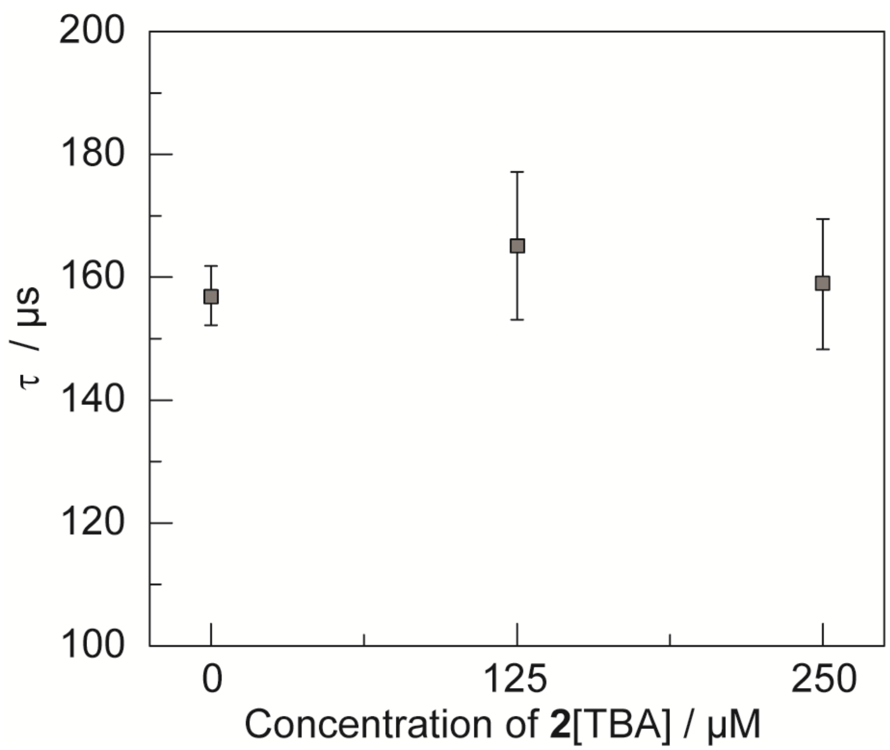

Figure S18. The lifetime, τ of the diphenylphosphinyl radical as a function of concentration of Ni complex **2**[TBA] concentration measured by nanosecond laser flash photolysis.

I. Turnover Frequency (TOF) for H_2 Production in Different Solvents

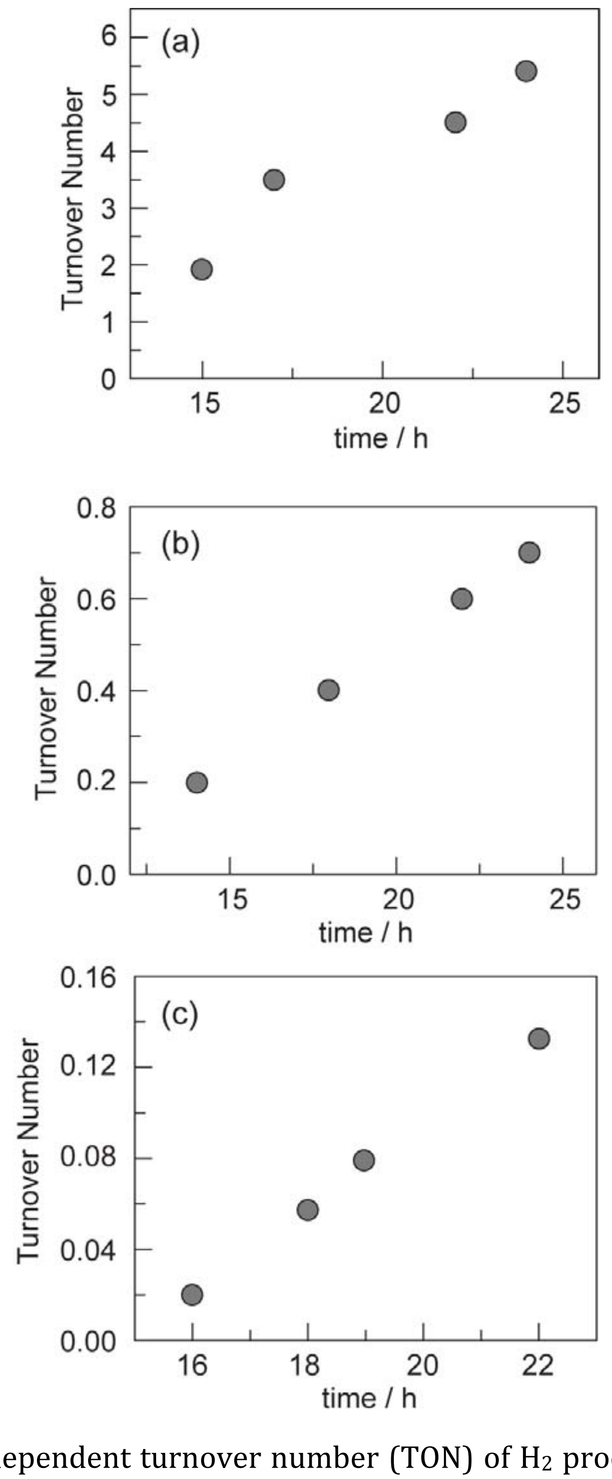

Figure S19. (a) Time dependent turnover number (TON) of H_2 produced by photolysis of: (a) **2**[TBA] in THF (λ > 295 nm) in the presence of 15 equiv of HCl; (b) **2**[TBA] in CH_3CN (λ > 295 nm) in the presence of 15 equiv of HCl; and, (c) **2**[TBA] in C_6H_6 (λ > 295 nm) in the presence of 15 equiv of HCl.

J. Electrochemical Data

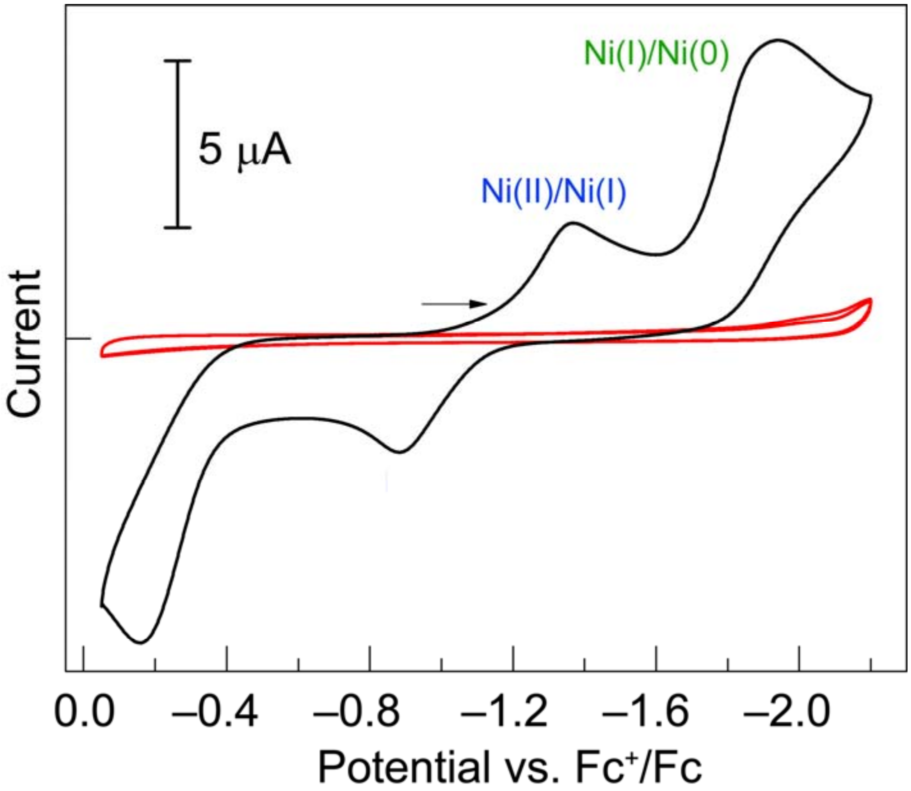

Figure S20. Cyclic voltammetry of 1 mM Ni complex **3** (▬, black) and solvent background (▬, red) measured with 0.1 M $^{n}Bu_4PF_6$ as a electrolyte in THF solution with a scan rate of 100 mV/s. Glassy carbon working electrode, Ag/$AgNO_3$ reference, and Pt wire counter electrode were used. E_p = –1.368 V, (Ni^{II}/Ni^{I}) and –0.885 V, (Ni^{I}/Ni^{II}).

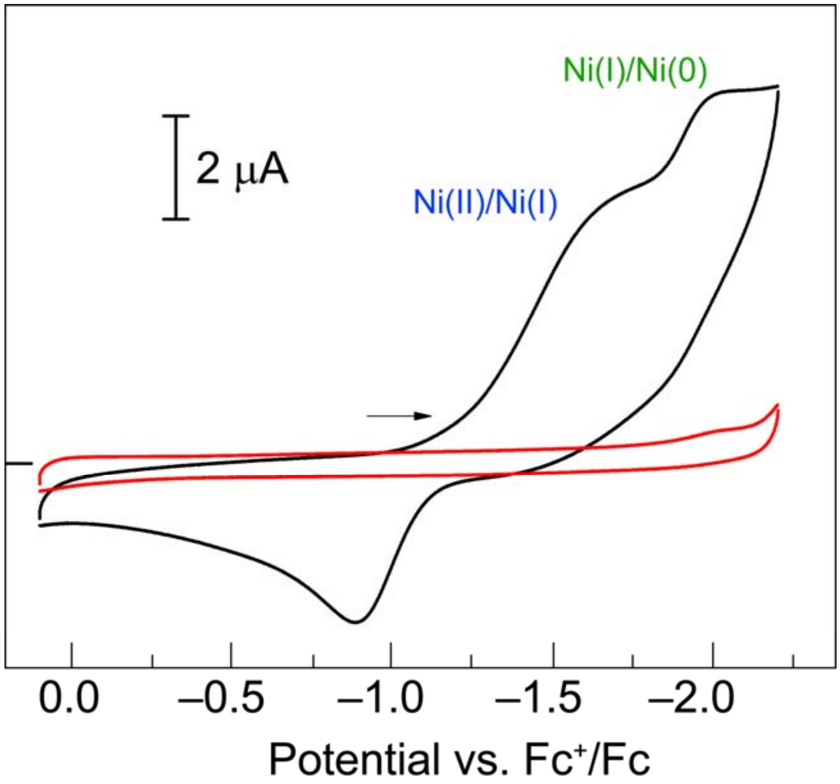

Figure S21. Cyclic voltammetry of 1 mM Ni complex **2**[TEA] (▬, black) and solvent background (▬, red) measured with 0.1 M $^{n}Bu_4PF_6$ as a electrolyte in THF solution with a scan rate of 100 mV/s. Glassy carbon working electrode, Ag/$AgNO_3$ reference, and Pt wire counter electrode were used. E_p = –1.628 V, (Ni^{II}/Ni^{I}) and –0.892 V, (Ni^{I}/Ni^{II}).
